# Promotion of Knowledge and Trust Surrounding Scarce Resource Allocation Policies: A Randomized Clinical Trial

**DOI:** 10.1001/jamahealthforum.2024.3509

**Published:** 2024-10-18

**Authors:** Russell G. Buhr, Ruby Romero, Lauren E. Wisk

**Affiliations:** 1 Division of Pulmonary and Critical Care Medicine, David Geffen School of Medicine at the University of California, Los Angeles; 2 Center for the Study of Healthcare Innovation, Implementation, and Policy, Health Services Research, Greater Los Angeles Veterans Affairs Healthcare System, Los Angeles, California; 3 Department of Health Policy & Management, Fielding School of Public Health at the University of California, Los Angeles; 4 Division of General Internal Medicine & Health Services Research, David Geffen School of Medicine at the University of California, Los Angeles

## Abstract

**Importance:**

The COVID-19 pandemic prompted rapid development of scarce resource allocation policies (SRAPs) in case demand for critical health services eclipsed capacity.

**Objective:**

To test whether a brief educational video could improve knowledge of how the University of California Health’s SRAP would be implemented and trust in health systems to implement such policies in accordance with ethical principles during the pandemic.

**Design, Setting, and Participants:**

This randomized clinical trial used an educational video intervention embedded in a longitudinal web-based survey and was conducted between May and December 2020 and analyzed during March 2024. A total of 1971 adult participants were enrolled, of whom 939 completed follow-up; participants with matched baseline and follow-up responses were analyzed. California residents were randomized to view the intervention (n = 345) or not (n = 353) and stratified by age, sex, education, racial identity, and self-reported health care worker status. Non-California residents were allocated to the control group (n = 241).

**Interventions:**

A brief (6-minute) “explainer” video that provided an overview of mechanics and ethical principles underpinning the University of California Health SRAP, subtitled in 6 languages.

**Main Outcomes and Measures:**

Self-reported survey assessment of knowledge of components of SRAP, graded as correct vs incorrect, and trust graded on a 10-point Likert scale. Anxiety about such policies was graded on a 10-point Likert scale with an a priori noninferiority margin of half of a standard deviation. Participants answered items at baseline and follow-up (approximately 10 weeks after baseline), with randomization occurring between administrations.

**Results:**

Of 770 randomized participants with responses at both points, 566 (73.5%) were female, and the median (IQR) age was 43.5 (36-57) years. Intervention participants demonstrated improvement of 5.6 (95% CI, 4.8-6.4; *P* < .001) more correct knowledge items of 20 vs controls, as well as significant improvements in reported trust in fairness/consistency and honesty/transparency about SRAP implementation. There was no significant change in reported anxiety surrounding SRAP in either treatment or control groups.

**Conclusions and Relevance:**

The trial found that a brief educational video is sufficient to explain complex ethical tenets and mechanics of SRAP and improved knowledge of such policies and trust in health systems to implement them equitably while not exacerbating anxiety about potential policy implications. This informs practice by providing a framework for educating people about the use of these policies during future situations necessitating crisis standards of care.

**Trial Registration:**

ClinicalTrials.gov Identifier: NCT04373135

## Key Points

**Question:**

Can a brief, unsupervised video intervention improve knowledge and trust of scarce resource allocation policies without increasing anxiety or fear on the topic?

**Findings:**

In this randomized clinical of 1971 adult participants randomized to view a brief explainer video or to a control group that did not view the video, there was an ability to significantly improve knowledge of the ethical frameworks and logistics of applying scarce resource allocation policy during health care crises, such as a pandemic. At the same time, trust in systems was significantly improved, and personal anxiety or concern felt when considering such policies did not worsen.

**Meaning:**

The trial results suggest that brief video interventions are an efficacious way to improve knowledge and trust of complex health policy topics and could potentially be useful across other health policy or operations issues.

## Teaser

This randomized clinical trial examines the efficacy of a brief educational video in improving knowledge of and trust in scarce resource allocation policies.

## Introduction

The COVID-19 pandemic put tremendous strain on health care delivery systems. The strain and concern that demand for health care could outpace supply, particularly for critical care resources like mechanical ventilators, led health systems and government bodies to promulgate scarce resource allocation policies (SRAPs) to triage access to intensive care. Such policies serve as a pressure release valve, guiding health workers in making systematic determinations about who should receive scarce resources based on preestablished ethical principles and clinical decision algorithms.

In spring 2020, the University of California (UC) convened a Critical Care Bioethics Working Group of clinicians and ethicists to design SRAP for their health system (UC Health) and published guidance in June 2020.^1^ Within ideal circumstances, this body would have also engaged community members and other key informants in the drafting of such a policy,^2,3,4,5,6,7,8,9^ but the urgency of the pandemic precluded their inclusion in the process. Because of this, UC Health sought community and health worker understanding and agreement with their interim policy by chartering the Understanding Community Considerations, Opinions, Values, Impacts, and Decisions (UC-COVID) study.^10^ This study evaluated opinions of a social media and community engagement–recruited sample of health workers and lay persons to provide feedback on components of the UC Health SRAP.^11^

Communication of science and public health messaging during COVID-19 was often fraught and contributed to confusion and distrust in health care and government authorities.^12,13^ It was expected that scare resource allocation algorithms would be controversial but potentially necessary policies during the pandemic. Clear health professional to patient communication is critical to empower ethical decision-making.^14^

Additionally, SRAP was covered in lay media, referencing moral distress and concern among those interviewed.^15,16,17,18,19,20,21^ UC Health recognized that optimal dissemination of SRAP would be needed to allay concerns and promote awareness of the policies and trust in the people and institutions that would need to execute them during a crisis. As such, a substudy was embedded within UC-COVID to test an educational intervention and assess whether knowledge and trust could be improved with a brief video designed to teach participants about the UC Health SRAP. The intervention sought to borrow techniques from established evidence on the communication of science and health policy and create educational support for an otherwise complex bioethics topic.^22,23^

In this article, we detail the findings of a randomized clinical trial (RCT) and its implications for health systems and governments preparing for potential crises. We demonstrate that such a tool can improve understanding and trust during times of crisis without increasing anxiety around such policies.

## Methods

### Recruitment

Detailed eligibility and recruitment methods for the UC-COVID study were previously published (Supplement 1).^10^ Briefly, any adult 18 years or older was eligible. We recruited via snowball sampling through social media and community partner referrals. A total of 1971 participants consented and enrolled in the baseline survey between May 8, 2020, and September 24, 2020. All were invited for follow-up and sent up to 3 reminder emails between August 10, 2020, and November 20, 2020, the prespecified recruitment period. The survey was conducted initially in REDCap (Vanderbilt University)^24,25^ and subsequently migrated to QualtricsXM (Qualtrics, Inc) for multilingual support. Ethics approvals were granted by the University of California Los Angeles institutional review board, and all participants provided electronic informed consent. Reporting followed the EQUATOR Network guidelines for RCTs of nonpharmacologic interventions.^26^

### Intervention Design

Our intervention was a 6-minute “explainer” video (Video) covering the mechanics and ethical principles underpinning the UC SRAP.^1^ An outline of the salient points that were most important for those who may need to implement SRAP or would be affected as patients was drafted by the Critical Care Bioethics Working Group. A script drafted at a sixth grade reading level (R.G.B. and L.E.W.) was furnished to a production studio (WorldWise Production) who animated it with a voiceover in English and subtitles in Spanish, simplified Chinese, Vietnamese, Korean, and Tagalog (the top 5 non-English languages spoken in California [the video was translated by International Contact]). The video introduced the SRAP, outlined the circumstances of its use, and explained the ethical principles and rationale underpinning the policy, the logistics of how the SRAP would function, consequences of nonallocation, and patients’ rights.

**Video.** Educational Video Describing a Scarce Resource Allocation Policy (SRAP) This video, shown to trial participants randomized to the intervention detailing SRAP, details the circumstances that may require SRAP implementation as well as the ethical and practical principles guiding its application.

### Randomization

Participants who completed a baseline survey were invited for follow-up and told they may be randomized to view an informational video. The text of each individual survey item and the explanatory headers briefly outlining SRAP can be found in eTable 1 in Supplement 2. Continuing participants were allocated a priori to the control arm if they self-identified as not being California residents at baseline, as UC Health SRAP is specific to California, to avoid confusing residents of other states whose policies may differ.

California residents were randomized using a parallel randomization algorithm (Qualtrics XM) at a 1:1 ratio as stratified by self-reported gender (female compared with all others), health care professional (HCP) occupation, education (<Bachelor’s degree compared with ≥Bachelor’s degree), race (American Indian/Alaska Native, Asian/Pacific Islander, and Black individuals compared with White individuals), Hispanic ethnicity, and age (<35, 35-55, and >55 years). Intervention participants viewed the brief video and completed follow-up survey items; control participants proceeded directly to the follow-up without the video. Investigators were masked to randomization until the final analysis.

### Primary Outcome

Knowledge evaluation items on policy logistics were operationalized as “This is true,” “This is false,” or “I’m not sure.” Items on critical care allocation were operationalized as “would be less likely/would be more likely/would not influence decisions about life support” or “I’m not sure.” A correct response was one matching the UC SRAP.^1^ The same items were asked at each point.

### Secondary Outcomes

Policy-related trust and anxiety items used 10-point Likert scales (strongly disagree to strongly agree). Intervention participants were additionally asked to provide feedback on the video regarding clarity, usefulness, and anxiety or unanswered questions surrounding the SRAP after the video using the same scaling.

### Missing Data

We analyzed those who responded to at least 1 item in each survey; some did not answer every question in a scale. Multiple imputation by fully conditional specification^27^ with 10 imputed datasets was used when a participant answered at least 1 item within a scale (eTable 1 in Supplement 2). Models were fit using imputed datasets and combined using Rubin rules.^28,29^ As estimates were consistent between imputed and nonimputed models, we reported imputed data as a sensitivity analysis to nonimputed main results.

### Statistical Analysis

We performed an intention-to-treat analysis. Responses were collated and paired within participants. We previously determined a sample of 172 total participants would detect a half SD change^30^ in correct knowledge items between groups with power of 90%.

We used randomization and an unexposed control because of the prevalence of media attention surrounding the SRAP during the time of administration to account for the net effect of the intervention and control for possible knowledge bleed-in from other sources. We used regression models to measure the pre/post change in the intervention group relative to pre/post change in the control groups. Random intercepts were included to control for repeated measures within participants. A Poisson regression was used for counts and fractional regression for the proportion of correct knowledge items, respectively. Linear regressions were used for changes in Likert ratings. Marginal estimates from regressions were used to calculate treatment effects and generate figures.^31^ Significance was determined using Wald tests, with Bonferroni adjustments for multiple comparisons. For anxiety items, we prespecified a noninferiority threshold of a half SD from pooled baseline responses.^30,32^ All tests were 2-tailed, with an α of .05. All analyses were conducted in Stata, version 18.0 (StataCorp).

## Results

### Recruitment

**Figure 1.**
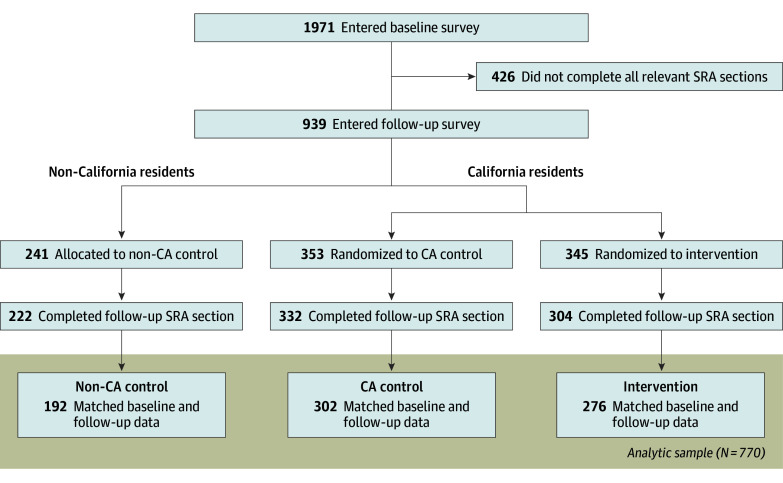
CONSORT Diagram Demonstrating Participant Flow and Randomization SRA indicates scarce resource allocation.

Of 1971 participants, 939 (48%) entered the follow-up survey between August 11, 2020, and December 5, 2020 (eFigure 1 in Supplement 2). The median time between surveys was 76 days (IQR, 27-94), which was not significantly different between the California control (CA control) and intervention groups (63 vs 52 days; *P* = .07; eFigure 2 in Supplement 2). A total of 770 participants provided responses at both points for items of interest to this analysis. Participant flow is shown in Figure 1.

### Participant Characteristics and Randomization

Baseline characteristics are described in the Table. The median age of participants was 43.5 (IQR, 36-57) years. Most participants self-reported being White (604 [78%]), followed by Asian/Pacific Islander (93 [12%]) and Black (42 [6%]), while 94 (12%) reported Hispanic ethnicity. Most had attained at least a Bachelor’s degree (662 [86%]), and 240 (31%) reported being a current HCP. There were no significant differences in any of demographic characteristics used for randomization between non-California (non-CA controls) and CA controls, nor between those in the CA control and intervention groups.

**Table.** Participant Characteristics by Group Allocation

Characteristic	No. (%)			
	All participants (N = 770)	Non-CA control (n = 192)	CA control (n = 302)	CA treatment (n = 276)
Age, y				
>35	191 (24.8)	52 (27.1)	78 (25.8)	61 (22.1)
35-55	366 (47.5)	95 (49.5)	150 (49.7)	121 (43.8)
>55	213 (27.7)	45 (23.4)	74 (24.5)	94 (34.1)
Sex				
Female	566 (73.5)	140 (72.9)	219 (72.5)	207 (75.0)
Male or other response^a^	204 (26.5%)	52 (27.1%)	83 (27.5%)	69 (25.0%)
Race and ethnicity^b^				
American Indian/Alaska Native	13 (1.7)	2 (1.0)	6 (2.0)	5 (1.8)
Asian/Pacific Islander	93 (12.1)	27 (14.1)	34 (11.3)	32 (11.6)
Black	42 (5.5)	15 (7.8)	15 (5.0)	12 (4.3)
Hispanic ethnicity	94 (12.2)	21 (10.9)	39 (12.9)	34 (12.3)
White	604 (78.4)	141 (73.4)	237 (78.5)	226 (81.9)
Other race	20 (2.6)	7 (3.6)	6 (2.0)	7 (2.5)
Health care professional	240 (31.2)	63 (32.8)	96 (31.8)	81 (29.3)
Education level				
>Bachelor’s degree	108 (14.0)	21 (10.9)	42 (13.9)	45 (16.3)
≥Bachelor’s degree	662 (86.0)	171 (89.1)	260 (86.1)	231 (83.7)

^a^ This group included 12 respondents who reported "some other gender" within that category: 6 reported transmasculine or transmale, 2 nonbinary, 1 gender fluid, and 3 genderqueer.

^b^ Race and ethnicity were framed as “select all that apply” and may sum to more than 100%.

### Outcomes

#### Knowledge

We observed no difference in correct items at baseline nor follow-up between non-CA controls and CA controls for any knowledge domains (ie, overall, logistics, health factors, or social factors). As such, while we retained both control groups for transparency, we focused on comparisons between CA controls vs intervention (ie, those randomized).

**Figure 2.**
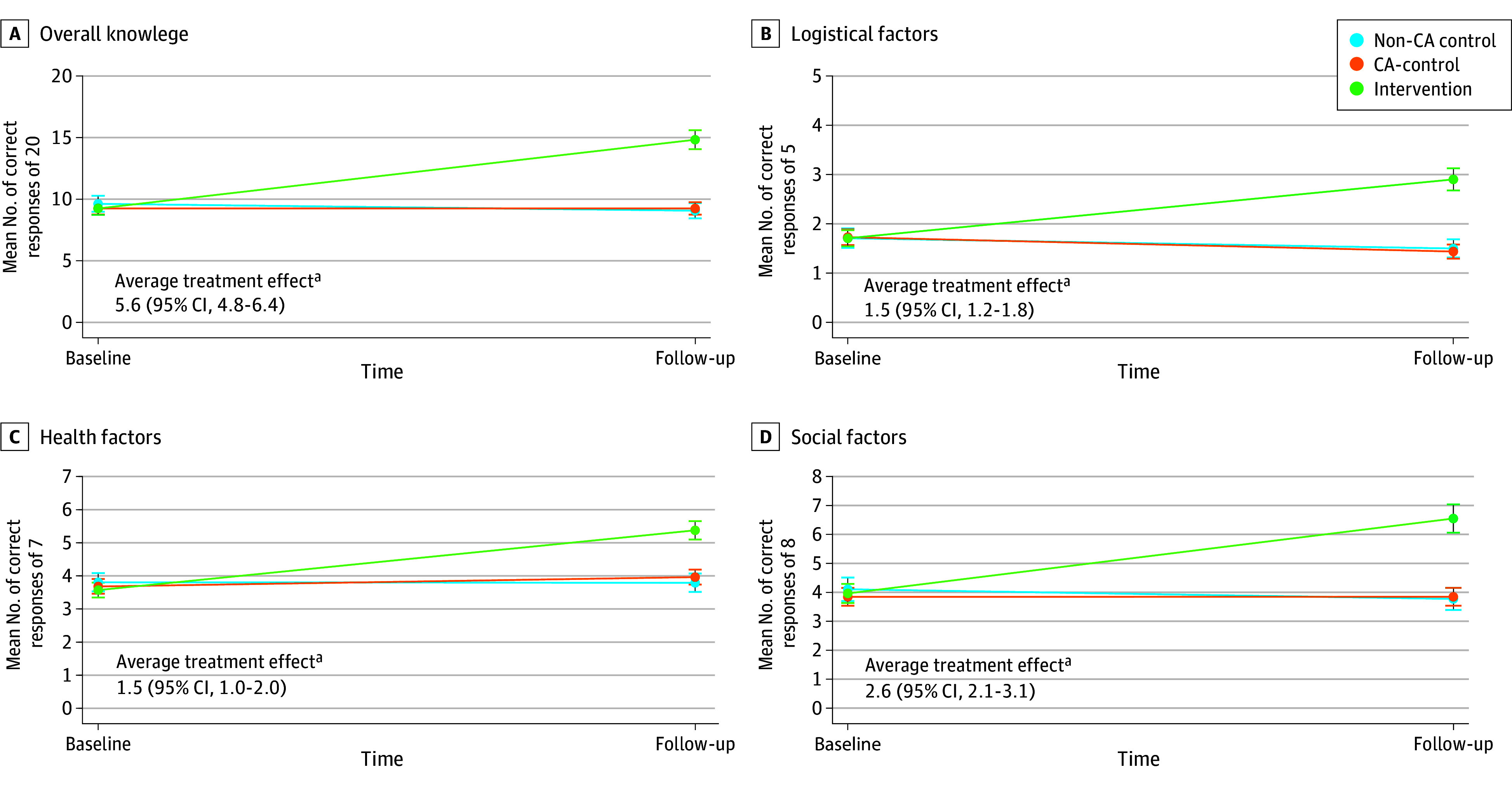
Marginal Estimates of Change in Correct Knowledge Items by Point and Randomization Group Indicators denote point estimates of group means with 95% CIs. Difference in differences between intervention and controls (average treatment effect) with CIs are shown. ^a^Significant at *P *< .001.

For overall knowledge, the primary end point, there was an improvement of 5.6 (95% CI, 4.8-6.4) more correct items within the intervention group compared with CA controls, a significant difference-in-differences (DID; *P* < .001; Figure 2; a full accounting of individual estimates can be found in eTable 3 in Supplement 2). This corresponded to 14.8 items (74.2%) correct for intervention vs 9.3 (46.3%) for controls (*P* < .001; eFigure 3 in Supplement 2), an absolute 28% point correct improvement (95% CI, 24.0%-31.9%; *P* < .001; eTable 4 in Supplement 2).

We found similar results for subdomains. On logistics, we observed 1.5 more correct items (95% CI, 1.2-1.8; *P* < .001) in the intervention, representing a 29.7% (95% CI, 25.0%-34.5%) percentage point improvement. On health factors, those who received the intervention improved by 1.5 more correct items (95% CI, 1.1-2.0; *P* < .001), representing a 21.7% (95% CI, 17.1%-26.3%) greater improvement. Regarding social factors, intervention participants answered 2.6 more correct items (95% CI, 2.1-3.1; *P* < .001), a 32.3% (95% CI, 26.5%-38.1%) percentage point improvement. Improvement for individual knowledge items was statistically significant for every item surveyed, with a range of improvement of 10.4% to 46.1% (eTable 2 in Supplement 2).

#### Trust

**Figure 3.**
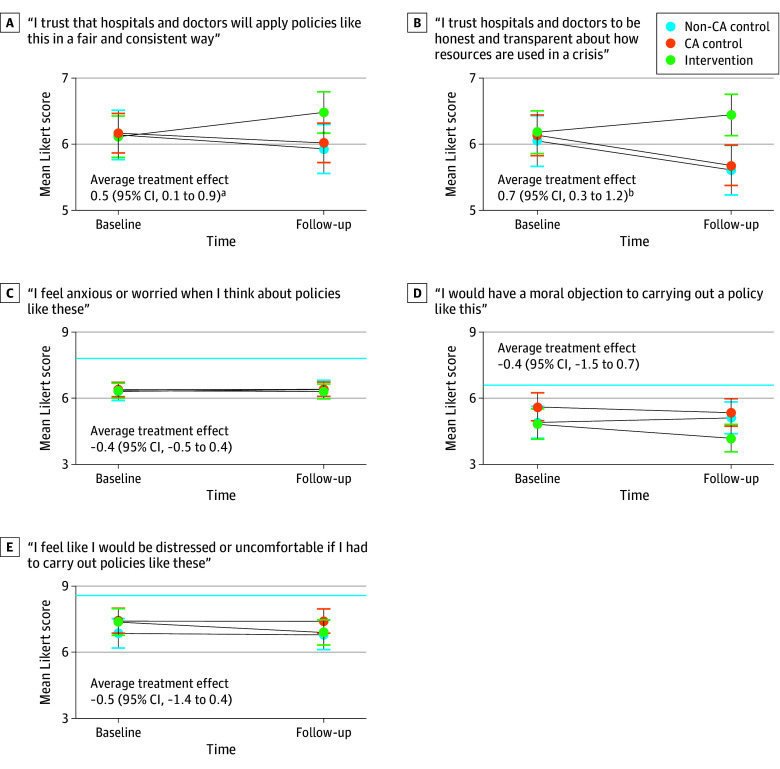
Marginal Estimates of Change in Trust and Anxiety Items by Point and Randomization Group The solid blue line indicates noninferiority threshold when appropriate. Indicators denote point estimates of group means with 95% CIs. Difference in differences between intervention and control (average treatment effect) with CIs are shown. A score of 1 indicates strongly disagree; a score of 10 indicates strongly agree. ^a^*P* < .05. ^b^*P* < .01.

When asked whether they trusted hospitals and physicians to apply SRAPs fairly and consistently, we found no significant difference between groups at baseline (Figure 3). At follow-up, we observed a significant DID of +0.5 Likert points (95% CI, 0.1-0.9; *P* = .02) toward agreement, with a mean (95% CI) postintervention agreement score of 6.5 (6.1-6.9) among those who received the intervention. In parallel, intervention recipients reported a significantly greater +0.7 Likert point (95% CI, 0.3-1.2; *P* = .002) agreement at follow-up as to whether they trusted that hospitals and physicians will be honest and transparent. The intervention group was associated with tighter, albeit small magnitude, correlation between knowledge and trust in fairness/consistency (Spearman ρ: 0.04 for control vs 0.11 for intervention) and transparency/honesty (ρ: 0.02 vs 0.12).

When asked whether they felt anxious about SRAP, no change was observed between intervention and control participants at either point. Those who self-identified as HCPs (228 participants) completed 2 additional items. Those in the intervention reported a change of −0.5 Likert points (95% CI, −1.4 to 0.4) in feeling that they would be distressed or uncomfortable if they had to implement an SRAP. When asked if they would have a moral objection to carrying out SRAPs, those in the intervention reported a change of −0.4 Likert points (95% CI, −1.5 to 0.7; eTable 5 in Supplement 2). No change in anxiety items crossed the noninferiority half SD margin.

#### Feedback on Intervention

**Figure 4.**
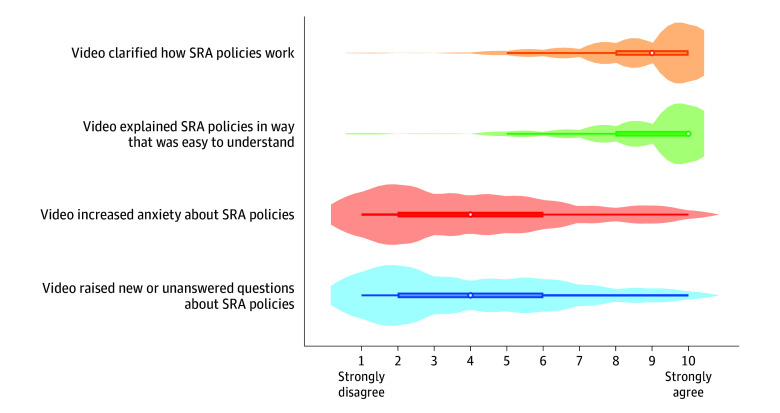
Respondent Feedback on Intervention’s Effect on Perception of Scarce Resource Allocation (SRA) Policies Medians denoted by white circles and interquartile ranges by colored bars, with the density of responses overlaid corresponding to the width of the violin.

Of participants who received the intervention, most reported that it clarified how an SRAP works, with a median agreement score of 9 of 10 (IQR, 8-10; Figure 4). Most also felt that the video was easy to understand (median, 10; IQR, 8-10). Corresponding to the main analysis, participants disagreed that the intervention increased their anxiety (median, 4; IQR, 2-6). Similarly, few were left with questions after viewing the video (median, 4; IQR, 2-6). No adverse events were reported during the study.

### Sensitivity and Stratified Analyses

When we added an interaction term to test the moderating effect of HCP status, improvement in knowledge was significantly greater among laypersons and HCPs overall and for each subdomain (eTable 6 in Supplement 2). Laypersons improved by a greater margin than HCPs (difference-of-differences-in-differences [DDD] of 1.2; 95% CI, 0-2.4; *P* = .04) on social factor knowledge. This was attributable to a greater baseline knowledge of social factors in SRAPs by HCPs. Laypersons and HCPs improved with intervention compared with controls for all other knowledge scales but did not have any significant differences in degree of improvement by HCP status.

Laypersons improved by a significant margin after intervention on whether they trusted hospitals and physicians to apply SRAPs in a fair and consistent way (DID, 0.6; 95% CI, 0.1-1.1; *P* = .03), while HCPs did not (DID, 0.3; 95% CI, −0.5 to 1.2), although the difference between these groups was negligible (DDD, 0.2; 95% CI, −0.7 to 1.2). Laypersons had a significant improvement in trust of hospitals and physicians to be honest and transparent (DID, 0.8; 95% CI, 0.2-1.3; *P* < .001), while HCP did not (0.6; 95% CI, −0.3 to 1.4]), again with no significant difference between groups (DDD, 0.2; 95% CI, −0.8 to 1.2). There were no significant changes in whether participants felt anxious or worried when thinking about SRAP in either group (eTable 7 in Supplement 2).

We also conducted stratified analyses across self-reported racial and ethnic groups, educational attainment, and age groups. We found a significant improvement in overall knowledge for every demographic group, with overlapping confidence intervals across levels for each variable, suggesting that similar effects were observed regardless of demographic characteristics (eTable 8 in Supplement 2). Participants who identified as Asian, were more highly educated, or younger scored the greatest improvement in trust items; no significant change in anxiety was reported in any subgroup (eTable 9 in Supplement 2).

We compared the observed responses with imputed data for missing items in scales. We found that knowledge gains in the imputed data showed an infinitesimally higher effect estimates for the intervention. For overall knowledge gains, this was a 0.052 (95% CI, 0.049-0.054) greater number of correct responses, with similarly small changes seen across knowledge subdomains and all trust or anxiety scale items (eTable 10 in Supplement 2).

## Discussion

In this RCT embedded within a web-based survey, we demonstrated that a brief educational video intervention was feasible to use even during the height of the COVID-19 pandemic, at a time when SRAP was not simply a hypothetical scenario, but a current and concrete possibility due to the existential threat that COVID-19 surges posed to health care system capacity. We focused on the immediate benefit of knowledge and trust, recognizing that SRAP is a time-sensitive issue for which patients need not have long-term knowledge improvements, but rather come to a quick understanding of an issue immediately affecting them. The intervention was well received and significantly improved knowledge of complex policy tenets and logistics, as well as trust in systems that would implement SRAP. While to our knowledge no known minimum clinically important difference thresholds have been published for these scales, statistically significant improvements were noted for all primary end points.

A major concern at the outset of the study was the potential for unintended harm from unsupervised learning on such a high-stakes topic, including increasing anxiety or fear about potential outcomes (eg, discontinuing life support against one’s wishes). We found that the intervention did not significantly provoke such feelings from participants, providing reassurance that an asynchronous video approach is acceptable for this application from a mental health and safety perspective.

Taken together, these clinical findings are uniquely relevant and provide insights on how institutions, systems, governments could invest in education and outreach about novel policies in a clinical setting to make sure that those potentially affected are well-informed about their effects.

Several studies have sought to understand values and preferences surrounding SRAPs across interested parties and groups,^11,33,34,35,36,37,38,39^ but to our knowledge, no currently published work has focused on knowledge and understanding. Similarly, while other studies have shown the utility of asynchronously delivered videos to influence health behaviors,^40,41^ to our knowledge no such initiatives have focused on policy education, with a recent systematic review citing an ongoing evidence gap.^42^ Therefore, this RCT demonstrates a significant gain not only in efforts to improve SRAP knowledge, but also in the field of patient and public education on health policy in general.

While knowledge in and of itself on SRAP may not be critical in abstract, improvement in understanding how policy functioned and increase in trust moved in parallel. Trust between patients and health care systems has eroded but remains critical in promoting health.^43,44,45^ Without trust, SRAP decisions are more likely to be met with resistance, regardless of the merits of the underpinning ethical frameworks, similarly to how mistrust of science and institutions increased COVID-19 vaccine skepticism.^46^ Similarly, failures of communication can foment distrust of recommendations or decisions surrounding life-sustaining treatment.^47,48^ Conversely, transparency on what patients can expect and promulgation of uniform standards of care promote trust.^45,49^ In implementing any policy, particularly on life-or-death decision-making, trust from affected parties is top priority.^50^

To our knowledge, no prior publications have addressed trust in SRAP. We found strong preferences for hospitals to make policy information public in a prior analysis.^11^ This paralleled other work showing that clear communication from a trusted authority with demonstrable expertise in a field is associated with improved acceptance of disseminated information.^49^ In this study, while we did not directly assess trust in the policy itself, trust in systems to carry out decisions with fairness and transparency was significantly improved by instruction on how the SRAP would work were it implemented. Taken together, the use of a concise, clear, and comprehensive video to educate on SRAP proved to be an important tool.

We were interested in the effect of the intervention on laypersons (potential patients) and HCPs who may need to implement such policies. In sensitivity analyses, we found that the effect of this intervention was durable across both of these groups, with a slightly greater effect among laypersons in knowledge improvement. We attribute this to HCPs being more likely to have some baseline knowledge and familiarity with SRAP from their day-to-day employment than those not in the health care sector. However, overall improvements in each group underscored that the comprehensibility of the information presented was acceptable to a wide range of audiences.

### Limitations

This intervention focused on SRAPs as developed by UC Health. Significant heterogeneity among such policies exist; thus, interventions would need tailoring across jurisdictions.^51^ While the video intervention was hosted on a private server and would therefore not have been possible for control participants to have access to it, it is still possible that they independently read about SRAPs on their own, diluting the observable effect of the intervention. If this were the case, the effects may be underestimated.

While the Hawthorne effect could have contributed, the randomization process helped to mitigate this bias. Additionally, we cannot fully exclude social desirability bias in responses, particularly around trust and anxiety. However, the survey was anonymous, and the spread of responses suggests that this was unlikely. As noted in prior publications, participants were recruited through snowball sampling and therefore not necessarily representative of the entire population, particularly notable in our sample being almost 75% women with less racial and ethnic diversity and a higher education level (due in part to the enrichment of recruiting HCPs) than the general population of California.^10^ However, other social media recruitment strategies for surveys have demonstrated adequacy.^52,53^

## Conclusions

In this randomized clinical trial, receipt of a brief educational intervention in the form of an animated explanatory video significantly improved knowledge and trust level surrounding SRAPs that may be implemented during a disaster-related or pandemic-related crisis. This represents a tool to rapidly educate those who may be affected by new policies or programs and is an area fertile for additional study similar interventions across different environments, groups, and policies.

## Notes

Supplement 1. Trial Protocol

Supplement 2. **eFigure 1.** Distribution of timing of survey responses **eFigure 2.** Days between survey administrations **eFigure 3.** Marginal estimates of percentage of correct responses to knowledge items by randomization group and time point by domain **eTable 1.** Questions from SRA survey with sampling universe **eTable 2.** Individual item mean scores by treatment group and time **eTable 3.** Marginal estimates for correct knowledge responses (main analysis) **eTable 4.** Marginal estimates for % correct knowledge responses (main analysis) **eTable 5.** Marginal estimates for trust and anxiety items (main analysis) **eTable 6.** Marginal estimates for correct knowledge responses by self-reported health care professional status (sensitivity analysis) **eTable 7.** Marginal estimates for trust and anxiety items by self-reported health care professional status (sensitivity analysis) **eTable 8.** Overall knowledge improvement treatment effect – stratified analyses by demographics **eTable 9.** Trust and anxiety item treatment effect – stratified analyses by demographics **eTable 10.** Comparison of imputed and unimputed datasets on outcomes

Supplement 3. Data sharing statement
