# Supplementary material for: Promotion of Knowledge and Trust Surrounding Scarce Resource Allocation Policies: A Randomized Clinical Trial
Source: JAMA Health Forum. 2024 Oct 18;5(10):e243509. doi: 10.1001/jamahealthforum.2024.3509 (PMC11489882; doi:10.1001/jamahealthforum.2024.3509)
Supplement: Supplement 1. — Trial Protocol [file jamahealthforum-e243509-s001.pdf]

Date: Friday, March 15, 2024 6:30:23 PM

Print Close

ID: IRB#20-000683 View: NEW 1.1 - Study Title and Key Personnel

This view has been locked by amendment(s)

Warning: Save your work at least every 15 minutes by clicking "Save" or "Continue."

Study Title and Key Personnel

All items marked with a red asterisk (*) are required. Items without an asterisk may or may not be required depending on whether the items are applicable to this study.

1.0 ***Full Title of the Submission:**
UC-COVID Study - Understanding Community Considerations, Opinions, Values, Impacts and Decisions for COVID-19

1.1 **Protocol Version Date and/or Number:**
2020-04-29 v2

2.0 ***Working or Lay Title:**
UC-COVID Study

3.0 **Principal Investigator:**

3.1 ***Name:** Russell Buhr
If the Principal Investigator requires a PI letter of exception, you are required to obtain one. More information can be found under UCLA Policy 900.
Degree(s): If degrees are not shown here, please add them to the next section, Section 1.1a/Item 1.0, which will then update the Principal Investigator's webIRB account information.
MD, PhD

3.2 **UCLA Title:** Assistant Professor-in-Residence

3.3 ***Will the Principal Investigator conduct the informed consent process with potential study participants?**

- (selected) **Yes**
- No
- Not Applicable

3.4 ***Is the Principal Investigator an undergraduate student, graduate student, post-doctoral fellow, or resident physician?**
Yes (selected) **No**

3.4.1 **If you answered "yes" to the above question, indicate the Faculty Sponsor for this study.**

The Faculty Sponsor must meet the requirements of UCLA Policy 900.

4.0 **Study Contact Person: Indicate the person, in addition to the Principal Investigator, who should receive all of the study correspondence.**
Lauren Wisk

5.0 **List the key personnel and study staff below.**

Note: All personnel listed below are required to complete CITI training courses (except for Fund Managers and Regulatory Coordinators). Please verify CITI training completion for all personnel prior to submitting a New Study application or Amendment application to add personnel. Verify using the Training Log tab in the application workspace (accessible by clicking the Exit button at the bottom of this page). HIPAA training is also required if personnel will be accessing protected health information.
Please make sure to have all personnel update their webIRB profile and contact information. Instructions on how to update the webIRB profile are available here.

	Name	Department	Role	Other Role (if applicable)	Will Obtain Consent?	Manage device accountability?	Access to personally identifiable info?	Access to code key?
View	Lawrence Benjamin	MEDICINE-PULMONARY DISEASE	Co-Investigator		no	Not Applicable	Yes	Yes
View	KAYLA BROWN	MEDICINE-DEPT ADMINISTRATION	Fund Manager (CITI not required if only serving in this role)		no	Not Applicable	No	No
View	JESSICA CHANNICK	MEDICINE-PULMONARY DISEASE	Co-Investigator		yes	Not Applicable	Yes	Yes
View	ROCHELLE DICKER	SURGERY-GENERAL	Co-Investigator		no	Not Applicable	No	No
View	I Obi Emeruwa	MEDICINE-PULMONARY DISEASE	Co-Investigator		no	Not Applicable	No	No
View	Estelle Everett	MEDICINE-ENDOCRINOLOGY	Co-Investigator		no	Not Applicable	No	No
View	Debra Faigh	MEDICINE-PULMONARY DISEASE	Fund Manager (CITI not required if only serving in this role)		no	No	No	No
View	Jane Fazio	MEDICINE-PULMONARY DISEASE	Co-Investigator		no	Not Applicable	Yes	Yes

View	Nicholas Jackson	MEDICINE-GENERAL MEDICINE & HLTH SRVCS.	Statistician or Data Analyst	no	Not Applicable	No	No
View	Hollyann Loui	MEDICINE-PULMONARY DISEASE	Co-Investigator	no	Not Applicable	Yes	Yes
View	CAROL MANGIONE, MD	MEDICINE-GENERAL MEDICINE & HLTH SRVCS.	Co-Investigator	no	Not Applicable	No	No
View	FOLASADE MAY	MEDICINE-GASTROENTEROLOGY	Co-Investigator	no	Not Applicable	No	No
View	MICHAEL ONG, MD, PhD	MEDICINE-VA WADSWORTH MED CTR	Co-Investigator	no	Not Applicable	No	No
View	NIDA QADIR	MEDICINE-PULMONARY DISEASE	Co-Investigator	no	Not Applicable	No	No
View	ALLISON RAMSEY	MEDICINE-PULMONARY DISEASE	Co-Investigator	yes	Not Applicable	No	No
View	NICHOLAS REPACI	MEDICINE-PULMONARY DISEASE	Fund Manager (CITI not required if only serving in this role)	no	Not Applicable	No	No
View	Ruby Romero	MEDICINE-GENERAL MEDICINE & HLTH SRVCS.	Research Assistant	no	Not Applicable	No	No
View	Lauren Wisk	MEDICINE-GENERAL MEDICINE & HLTH SRVCS.	Co-Principal Investigator	yes	Not Applicable	Yes	Yes
View	Caitlin Yumori	MEDICINE-PULMONARY DISEASE	Research Assistant	no	Not Applicable	No	No

ID: IRB#20-000683 View: NEW 1.1a - Other Personnel

This view has been locked by amendment(s)

Warning: Save your work at least every 15 minutes by clicking "Save" or "Continue."

Other Personnel

All items marked with a red asterisk (*) are required. Items without an asterisk may or may not be required depending on whether the items are applicable to this study.

1.0 Principal Investigator

1.1 **Name:** Russell Buhr
*__Please type the Degree(s):__ MD, PhD

1.2 **Principal Investigator's UCLA Department:** MEDICINE-PULMONARY DISEASE

1.3 *__Protocol's UCLA Home Department:__ MEDICINE-PULMONARY DISEASE

This response defaults to the PI's payroll department. If you wish to affiliate this protocol with another department, please select the department from the list above.

For tips on effective search, please see guidance to the right.

2.0 If there will be other types of personnel working directly under the PI's supervision on aspects of the study, provide their name, title and institution, indicate their responsibilities, training and qualifications and complete Item 2.1.

Please also indicate, if applicable, whether that person will obtain consent, manage device accountability, have access to personally identifiable information and/or have access to the code key.

Please use a new entry to add each individual unless describing a class of individuals who rotate through the study team (see guidance area to the right).

Note: If there will not be other types of personnel go to Item 3.0.

	Name, title, institution	Study role(s): e.g., conduct interviews/surveys, recruit participants, obtain consent, review records, etc.
View	Dominic Mangino, UCLA medical student	Will assist with data cleaning and analysis, recruiting to and conducting focus groups Student not listed in UCLA WebIRB
View	UCLA Student Research Program/undergraduate students	Students will assist with data cleaning and analysis, recruiting subjects, and conducting focus groups. They will complete the required CITI human subjects and HIPAA training as outlined below. They will be supervised by Dr. Wisk and Dr. Buhr directly.

For existing protocols: Item 2.0 has been modified and this item cannot be edited. When submitting an amendment please use the information found in the text box below to complete Item 2.0 above.

Briefly describe the other study personnel.

2.1 **Indicate the human subjects research training these personnel have or will receive. If training is required in a language other than English or if research is occurring in a location where research personnel do not have access to the internet (e.g., rural community without internet capability), please describe how human subjects training requirements will be fulfilled.**

Check all that apply:

- [x] **CITI Training**

- [x] **UC HIPAA Training**
- [ ] Other

2.2 **If you indicated "Other" to item 2.1, describe:**

2.3 *__Will this study use the UCLA Health Sciences Volunteer Program to assist with the conduct of the research study?__

( ) Yes (•) **No**

3.0 *__Will any of the study procedures or analyses be contracted to a consultant or an organization?__

( ) Yes (•) **No**

3.1 **If yes, specify the consultant(s) and/or organization(s) and the work that they will do for the study.**

ID: IRB#20-000683 View: NEW 1.1b - Type of Study Review

Warning: Save your work at least every 15 minutes by clicking “Save” or “Continue.”

Type of Study Review

1.0 ***Indicate the level of risk involved with this study.**

(if there are multiple groups or phases associated with this study, select the highest level of risk.)

(•) **Minimal risk or no known risks - Click here for the OHRPP tip sheet on minimal risk.**

( ) Greater than minimal risk

2.0 *__Indicate the type of review that you are requesting for this study.__

( ) IRB Review: Expedited or Full Board

(•) **Certification of Exemption from IRB Review**

2.1 **If you indicated “IRB Review: Expedited or Full Board” as the type of review in item 2.0, select the IRB that you think best matches your research.**

	Name	Description
( )	Medical Institutional Review Board 1	MIRB1 reviews general and internal medicine, infectious diseases and ophthalmologic research.
( )	Medical Institutional Review Board 2	MIRB2 reviews oncology and hematology research.
( )	Medical Institutional Review Board 3	MIRB3 reviews neuroscience, neurology, psychiatric, drug abuse and dental research.

◯	North General Institutional Review Board	NGIRB reviews research from the College of Letters & Science and the Professional Schools.
◉	**South General Institutional Review Board**	SGIRB reviews social-behavioral research from the Schools of Public Health, Nursing, and Medicine.

***Please note**: The above requests are for initial routing purposes only. The final decision as to committee assignment and type of review, rests with OHRPP and/or the IRBs.*

3.0 ***Is this a COVID-19 research proposal that falls under the following scope:**

a. **Access to the suspected and confirmed UCLA Health COVID-19 patients.**
b. **Access to the electronic medical record chart or data of those patients.**
c. **Access to the remnant or research biospecimen collection of those patients.**
d. **Planning any clinical research interventional trial (drug/device) for those patients.**
e. **COVID Population-based studies that overlap the UCLA Health population or UCLA healthcare workers.**

◉ **Yes**
◯ No

ID: IRB#20-000683 View: NEW 1.2 - Conflict of Interest Information

Warning: Save your work at least every 15 minutes by clicking "Save" or "Continue."

Conflict of Interest Information

1.0 *** Does the Principal Investigator, any of the key personnel, or their spouses, registered domestic partners, or dependent children, have a financial interest in the sponsor (profit, non-for-profit) of the research?**

◯ Yes ◉ **No**

1.1 **If yes, attach a completed copy of the Financial Interests Form for each person who indicates a financial or related interest:**

Document Name	Document Version #
There are no items to display	

2.0 *** Does the Principal Investigator, any of the key personnel, or their spouses, registered domestic partners, or dependent children, have any financial interests related to the research sponsored by a government agency?**

◯ Yes ◉ **No**

2.1 **If yes, attach a completed copy of the Financial Interests Form:**

Document Name	Document Version #
There are no items to display	

3.0 * **Indicate whether any of these financial interests have been submitted to or reviewed by the UCLA campus Conflict of Interest Review Committee (CIRC):**

◯ Yes ◉ **No**

3.1 **If you have received a response from CIRC, attach it here:**

Document Name	Document Version #
There are no items to display	

ID: IRB#20-000683 View: NEW 1.3 - Study Locations

Warning: Save your work at least every 15 minutes by clicking "Save" or "Continue."

Study Locations

1.0 ***Indicate the locations where any research activities will be performed by the UCLA research team with participants and/or private information obtained.**

Check all that apply:

- ☐ a. UCLA Sites or UCLA Health System Sites (Does not include Harbor-UCLA Medical Center, Olive View-UCLA Medical Center, or Orthopaedic Institute for Children)
- ☐ b. Off Campus (in California)
- ☐ c. Outside California (in the U.S.)
- ☐ d. Outside the United States ***See note at right**
- ☑ **e. Internet**

1.1 **If you selected b, c or d above, please provide your assurance that documentation of each site's permission to conduct the research at the site(s) will be obtained and maintained by the UCLA PI as applicable:**

Agree ☐

2.0 ***Is this a multi-institutional study (i.e., a collaborative project with other sites that have their own IRBs or principal investigators)?**
(Includes but not limited to UC MOU and CTSI MOU collaborations where UCLA IRB review is requested.)

◯ Yes ◉ **No**

If no, please skip directly to the next page, do not complete the questions below.
If yes, please answer items 2.1-2.3:

2.1 **Will UCLA be responsible for the overall direction of the study at the other institutions?**

◯ Yes ◯ No

2.1.1 **Indicate the measures that will be taken to assure regulatory**

compliance at each site and that the following types of information will be communicated to the other sites: study procedures; modifications to the protocol and related documents; and safety updates, interim results and other information that may impact risks to study participants.

Check all that apply:

- ☐ Conference calls or meetings with minutes distributed to each site
- ☐ Timely e-mail communications
- ☐ Postings on the study website
- ☐ Other

2.1.1.1 If you chose "other", describe.

2.1.2 If you answered "yes" to item 2.1 above, please provide your assurance that the current IRB approval for each site(s) will be obtained and maintained by the UCLA PI as applicable:
Agree ☐

2.2 Will the UCLA principal investigator specified on this application be responsible for the data coordinating center?

2.3 Indicate the anticipated total number of study participants that will be enrolled across all of the institutions.

ID: IRB#20-000683 View: NEW 2.1 - Project Identification Information

Warning: Save your work at least every 15 minutes by clicking “Save” or “Continue.”

Project Identification Information

1.0 *Type of Submission (Select one)

- ◉ **Research Study**
- ○ Application for Approval of "Research Participant Pool" or recruitment database only

2.0 *Type of Submission (Select one)
For Amendments, do not undo the response below. Undoing the response may remove sections of the original application.

- ◉ **New Submission**
- ○ Transfer of Ongoing Research from Another Site from Investigator moving to UCLA. Please complete Item 2.1.

2.1 **If you selected "Transfer of Ongoing Research" in Item 2.0 indicate the current status of the study and a brief summary of the work to date.**

3.0 ***Who developed this study?**

Check all that apply:

- [x] **UCLA investigator**
- [ ] Investigator from another institution
- [ ] Industry/Pharmaceutical Company
- [ ] Cooperative Group (e.g., Children's Oncology Group, AIDS Clinical Trial Group)
- [ ] Other

3.1 **If other, specify.**

4.0 **Review For and Reliance Upon External IRBs.**

***Indicate if one of the following applies to this study. (Select one)**

- (•) **None of the options apply.**
- ( ) UCLA IRB to serve as IRB of record for another institution.

5.0 ***Is this study cancer related,** including the recruitment of individuals with cancer, collection of cancer human biological samples, specimens or data, or the recruitment of individuals because they are cancer survivors or at risk of developing cancer?

( ) Yes (•) **No**

Note: If you answered "Yes", you must submit an application to the Jonsson Comprehensive Cancer Center (JCCC) Internal Scientific Peer Review Committee (ISPRC). Click here for instructions for submitting to the ISPRC. The ISPRC approval notice or letter of exemption should be attached in Section 2.1/Item 7.2 of the webIRB application.

6.0 ***Nurse Involvement:** Does this study involve any nursing time, effort, and/or resources at UCLA Health System sites, including as subjects, investigators, clinical care providers or data or specimen collectors?

( ) Yes (•) **No**

Note: If you answer "Yes", please submit an application to the Research and Innovation Council (RIC). For more information about nursing review and how to apply, click here. ***IRB approval is not contingent on RIC approval and you do not need to upload documentation of approval from the RIC into webIRB.***

7.0 ***Federal regulations (45 CFR 46.111) require scientific review before an IRB approves a study. For the majority of studies being reviewed and approved by the UCLA IRB, the IRB performs this review.**
See http://ora.research.ucla.edu/OHRPP/Documents/Policy/4/Scientific_Review.pdf for additional details.

Do you want the IRB to consider external scientific or scholarly review?

◯ Yes ◉ **No**

7.1 **If yes, indicate the source of scientific or scholarly review for the study.**

Check all that apply.

- ☐ National Institutes of Health (NIH)
- ☐ The funding agency (other than NIH)
- ☐ Faculty Sponsor
- ☐ JCCC – Internal Scientific Peer Review Committee (ISPRC)
- ☐ Clinical Translational Research Center (CTRC)
- ☐ UCLA Department
- ☐ Other

7.1.1 **If you checked "other", describe.**

7.2 **Attach a copy of the scientific or scholarly review, if applicable.**

Document Name	Document Version #
There are no items to display	

ID: IRB#20-000683 View: NEW 2.2 - Lay Summary and Keywords

Warning: Save your work at least every 15 minutes by clicking "Save" or "Continue."

Lay Summary and Keywords

Please provide the following information about your study.

1.0 ***Provide a brief lay summary describing this study. (limit 500 words).**
The novel coronavirus (COVID-19) is affecting the way many people live their lives, including seeking medical care and maintaining good self-care to keep healthy. Additionally, in the event many people become critically ill at once, COVID-19 has the possibility of overwhelming hospitals to the point where they have to make decisions about how to determine who receives intensive care and life-support measures. Many hospitals as well as local or state governments have been working on policies to determine how to make these decisions. This study seeks to learn about how COVID-19 has affected the way patients and healthcare providers care for themselves and about their thoughts and concerns about policies that may "ration" life-support resources.

2.0 ***List three to five keywords describing this study (separate the words with commas). The keywords may be used for identifying certain types of studies.**
COVID19, rationing, scarce resource allocation, intensive care, health behaviors

3.0 *** Is this study conducted or supported by HHS (e.g., the National Institutes of Health, Centers for Control and Prevention, etc.)?**

◯ Yes ◉ **No**

4.0 * **Is this study regulated by the Food and Drug Administration (FDA)?**
◯ Yes ◉ **No**

4.1 **If yes, check all that apply:**

- [ ] Human Drugs
- [ ] Medical Devices
- [ ] Biological Products
- [ ] Mobile Medical Applications
- [ ] Food Additives
- [ ] Color Additives
- [ ] Other

ID: IRB#20-000683 View: NEW 2.3 - Methods/Procedures - Descriptors

Warning: Save your work at least every 15 minutes by clicking "Save" or "Continue."

Methods/Procedures - Descriptors

Note: The items listed below are not an inclusive list of methods and procedures that may be used in research studies. The list only includes items that will trigger additional questions related to the research or are needed for the review process

1.0 ***Indicate all that apply to this study.**

- [x] **Audio, Visual or Digital Recordings**
- [ ] Certificate of Confidentiality for research not supported by NIH (please see Quick Guide and Tip Sheet)
- [ ] Clinical Trial of a Drug, Biologic, Device or a Behavioral Intervention (complete item 3.0 below)
- [ ] Community Based Research
- [ ] Controlled Substances (Schedule I or II)
- [ ] Deception or Partial Disclosure
- [ ] Devices/Diagnostics (Note: Submit all HUDs in BruinIRB)
- [ ] Drugs/Biologics/Dietary Supplements
- [ ] Genetic Analyses/Genotyping
- [ ] Human Embryonic Stem Cells and/or Induced Pluripotent Stem Cells
- [ ] Human Gene Transfer/ Recombinant DNA
- [ ] Infectious Agents
- [ ] Non-FDA approved medical equipment used with UCLA hospital patients or research participants that operate under the UCLA Hospital License.
- [ ] Radiation (Standard of Care or Investigational Use of radioactive materials, radiation producing machines or ionizing radiation)
- [ ] Substance Abuse Research (with Medication)
- [ ] Treatment in an Emergency Setting (with request to waive consent)

- [ ] **None of the above**

2.0 ***Will the study require services or resources owned/rented/operated or provided by the UCLA Health System (e.g. clinic and/or hospital visit(s), CTRC, professional medical services, clinical treatment, diagnostics, labs, medical supplies, etc.)?**

Please direct any questions about this to The Financial Coverage & Activation Team at coverageanalysis@mednet.ucla.edu.

( ) Yes (•) **No**

3.0 ***Criteria to meet the NIH definition of a Clinical Trial (check all that apply):**

- [ ] Does the study involve human participants research?
- [ ] Are participants prospectively assigned to an intervention? NIH defines an “intervention” as a manipulation of the subject or subject’s environment for the purpose of modifying one or more health-related biomedical or behavioral processes and/or endpoints.
- [ ] Is the study designed to evaluate the effect of the intervention on the participants?
- [ ] Is the effect being evaluated a health-related biomedical or behavioral outcome? NIH defines a “health-related biomedical or behavioral outcome” as the pre-specified goal(s) or condition(s) that reflect the effect of one or more interventions on human subjects’ biomedical or behavioral status or quality of life.
- [ ] None of the above

ID: IRB#20-000683 View: NEW 6.1 - Funding and Other Study Characteristics

This view has been locked by amendment(s)

Warning: Save your work at least every 15 minutes by clicking “Save” or “Continue.”

Funding and Other Study Characteristics

1.0 ***Indicate the funding status for this study.**

( ) Funded

( ) Application for funding is pending

(•) **Departmental funding / Self funding / No funding**

2.0 ***Check all that apply**:

- [ ] The research will be conducted through the UCLA Clinical and Translational Research Center (CTRC)
- [ ] The study will be supported by or conducted in collaboration with the U.S. Department of Defense (DOD)
- [ ] The study will be supported by or conducted in collaboration with the U.S. Department of Energy (DOE)
- [ ] The study will be supported by or conducted in collaboration with the U.S. Department of Justice (DOJ)
- [ ] The study will be supported by or conducted in collaboration with the U.S. Department of Education (ED)
- [ ] The study will be supported by or conducted in collaboration with the U.S. Environmental Protection Agency (EPA)

☑ **None of the above**

2.1 **If you selected DOD, DOE, DOJ, ED, and/or EPA support/collaboration, please provide your assurances that you will review the additional requirements for research supported by the relevant federal agency.**
Agree ☐

Note: Please refer to the Federally-Supported Research section of the OHRPP guidance document: Funding Considerations for Federally-Funded and Industry-Sponsored Human Research.

ID: IRB#20-000683 View: NEW 8.1 - Study Design

Warning: Save your work at least every 15 minutes by clicking "Save" or "Continue."

Study Design

1.0 ***Check all that apply to the study design.**

☑ **Direct subject contact ONLY – The research activities involve direct contact with study participants (e.g., collection of data or specimens in person or via internet, phone, mail, etc.)**

☐ **No direct subject contact** – None of the research activities involve direct contact with study participants and include only analyses of data, records and/or human biological specimens (e.g., medical record or other record review, study of specimens left over from clinical procedures).

☐ **BOTH Direct subject contact AND No direct subject contact** – Some of the research activities involve direct contact with study participants and some of the research activities involve analyses of data, records and/or human specimens obtained without contact with participants.

ID: IRB#20-000683 View: NEW 8.8 - Audio, Visual or Digital Recordings

Warning: Save your work at least every 15 minutes by clicking "Save" or "Continue."

Audio, Visual or Digital Recordings

You indicated that this study includes recordings (audio or visual) (section 2.3/item 1.0). Please provide the following information.

1.0 ***Who will transcribe the research tapes/recordings?**

Check as many as apply:

☐ Members of the research team

☑ **Persons outside the research team**

2.0 ***Is the use of recordings an optional part of the research?**
○ Yes ● **No**

3.0 *** Will individual study participants be able to review, edit, and erase the tapes/recordings of their research**

participation?
Yes **No** (selected)

3.1 **If no, provide an ethical and scientific justification for NOT allowing study participants to review, edit, and erase the tapes of their research participation.**
Participation in focus groups is anonymous. It is not feasible to separate one indivdual's taped recording without destroying others' contributions.

4.0 Transcription of Research Tapes/Recordings

4.1 * **Type of media (Check as many as apply):**

- [ ] CD ROM
- [ ] DVD
- [x] **Digital Files**
- [ ] VHS tape
- [ ] Cassette or microcassette
- [ ] Handwritten files
- [ ] Other

4.2 * **Method of transmission (Check as many as apply):**

- [ ] Courier or mail with delivery confirmation
- [x] **Posted to a secure website**
- [ ] Email
- [ ] Other
- [ ] **Not Applicable**

4.3 * **Transcription Service (Check as many as apply):**

- [ ] Transcription service secures tapes in a secure locked area
- [x] **Transcription(s) sign confidentiality agreements**
- [x] **Transmission of voice files and text files is encrypted and password protected**
- [ ] Other
- [ ] **Not Applicable**

4.3.1 **If you selected "other" for any/all of the above items, describe.**

ID: IRB#20-000683 View: NEW 9.2 - Information about Study Data

This view has been locked by amendment(s)

Warning: Save your work at least every 15 minutes by clicking "Save" or "Continue."

Information about Study Data

This information is needed to determine how you will best protect the confidentiality of data.

1.0 ***Indicate all that apply to the study data.**

Check all that apply:

- [ ] Obtained from a medical or clinical record
- [ ] Created or collected as part of health or mental health care
- [ ] Used to make healthcare or mental healthcare decisions and/or provided to other healthcare professionals
- [ ] Research data will be entered into the participants' medical or clinical record
- [x] **None of the above**

2.0 ***Is it reasonably foreseeable that the study will collect information that State or Federal law requires to be reported to other officials (e.g., child or elder abuse), ethically requires action (e.g., suicidal ideation), or is a reportable disease?**

( ) Yes (•) **No**

2.1 **If yes, explain below and include a discussion of the reporting requirements in the consent document:**

3.0 ***Indicate if any of the following are being obtained and used without any direct contact with study participants.**

- [ ] Records (Not medical)
- [ ] Human biological specimens
- [x] **None of the Above**

4.0 *Indicate all identifiers that may be **accessed or included in the research records** for the study:

- [ ] Names
- [ ] Dates
- [x] **Age (if over 89 years)**
- [ ] Postal Address
- [ ] Phone Numbers
- [ ] Fax Numbers
- [x] **E-Mail Address**
- [ ] Social Security Number
- [ ] Medical Record Number
- [ ] Health Plan Numbers
- [ ] Account Numbers
- [ ] License/Certificate Numbers
- [ ] Vehicle ID Numbers
- [ ] Device Identifiers/Serial Numbers
- [ ] Web URLS
- [x] **IP Address Numbers**
- [x] **Biometric Identifiers (including finger and voice prints)**

Facial Photos/Images

- [x]
- [ ] Any Other Unique Identifier (this does not include the code assigned by the investigator to identify the data)
- [ ] **None of the above**

4.1 **If social security numbers will be collected explain why they are necessary, how they will be used, how they will be protected and how long they will be retained.**

5.0 ***Select all that apply:**

- [ ] The data and/or specimens will be directly labeled with personal identifying information when acquired by the investigator for this research
- [ ] The data and/or specimens will be labeled with a code that the research team can link to personal identifying information when acquired by the investigator for this research
- [x] **The data and/or specimens will not be labeled with any personal identifying information, nor with a code that the research team can link to personal identifying information when acquired by the investigator for this research**
- [ ] The data are restricted use data (A term used in Social-Behavioral research. See guidance on the right.)

5.1 **Indicate how the data will be used when this study is completed.**

Check all that apply:

- [x] **Use for this study**
- [x] **Use for possible future research**
- [ ] Use to create a bank or repository at UCLA
- [ ] Add to existing repository
- [x] **Other**

5.1.1 **If Other, specify:**
E-mail addresses submitted by the subjects will be retained for future surveys only if the subject grants permission to be contacted in the future (which is one of the specific questions in the instrument).

ID: IRB#20-000683 View: NEW 9.2a - Privacy and Confidentiality

This view has been locked by amendment(s)

Warning: Save your work at least every 15 minutes by clicking "Save" or "Continue."

Privacy and Confidentiality

Important Notes:

- **Privacy is about people.** Privacy refers to a person's wish to control the access of others to themselves.
- **Confidentiality is about data.** Confidentiality refers to the researcher's plan to handle, manage,

and disseminate the participant's identifiable private information.

See OHRPP Quick Guide: Protecting Privacy and Maintaining Confidentiality

1.0 ***Privacy: How will the investigator maintain privacy in the research setting(s)?**
(e.g., interviewing participant in a room or area where conversations cannot be overheard by others, or conducting medical procedures in an examination room, or behind a curtain in an emergency room).

Surveys: Participants will fill out the survey using internet-based collection over RedCAP or Qualtrics and can fill out the survey in a private location of their choosing.

Focus groups: Participants will be enrolled in focus groups by invitation from prior survey participation. Participants have the choice as to where they will choose to be during participating in the web-based focus group. They also will be able to choose to turn off or on their own video for capture and how to display their name.

Analysis of internet-based social media comments: Comments made on social media posts are made in the public domain and do not afford the reasonable assumption of privacy on the part of the poster if the person making the post has a public facing social media account.

2.0 ***Confidentiality: If the protocol will collect and maintain identifiable data, explain how the planned safeguards to maintain confidentiality of identifiable data and data security are appropriate to the degree of risk from disclosure.**

Note: Other sections of the application (e.g., Sections 9.3, 9.3a, 9.4, 9.5, and 15.3) will request specifications such as identification of persons who will have access to code keys or measures to comply with HIPAA requirements.

Surveys: IP and e-mail addresses will be necessarily captured in RedCAP or Qualtrics to link participants for repeat survey collection. These items are stored internally in RedCAP or Qualtrics and the investigators do not have direct access to these items. E-mail addresses are also collected in order to contact participants who are selected to receive honoraria through the lottery system and will not be stored directly with research data. Those addresses will not be used for any other purposes and will be destroyed after completion of the study.

Focus Groups: Focus groups will be conducted over encrypted connections using Zoom meetings with password protection. No personal information will be collected, however internet focus groups will be recorded for analysis. Only the investigators will have access to the video, which will be stored in an encrypted drive on a computer stored in a locked office in a secured suite. Audio files will be securely transferred without audio to the transcription service over an encrypted file transfer service. No personal identifying information other than voice print will be transmitted for transcription.

Analysis of internet-based social media comments: Comments made by individual users on social media platforms on news media articles, tweets, Facebook, LinkedIn, and Doximity posts will be collected, but without link to the profile of the person making the comment.

ID: IRB#20-000683 View: NEW 9.8 - Data and/or Specimens for Possible Future Use

Warning: Save your work at least every 15 minutes by clicking “Save” or “Continue.”

Data and/or Specimens for Possible Future Use

You indicated that prospectively collected data and/or specimens would be stored for future use (Section 9.2/item 5.1). Please provide the following information.

1.0 ***Specify what information directly or indirectly linked to the subject will be provided with data and/or specimens to other investigators.**

Check all that apply:

- [ ] No subject identifiers (The data/specimens are anonymous; no one including the investigator could identify the person from whom the materials were gathered.)
- [ ] The data will be coded (A code links the data/specimens to the study participants. A key to the code exists.)
- [ ] Personal Identifying Information
- [x] **Not applicable, the data will not be shared outside the study team.**

2.0 **Distribution Rules: Describe the criteria used to determine the adequacy of requests to obtain data and/or specimens (e.g., the type of researchers that will be eligible to receive data):**

ID: IRB#20-000683 View: NEW 10.1 - Study Summary - Research Study

This view has been locked by amendment(s)

Warning: Save your work at least every 15 minutes by clicking "Save" or "Continue."

Study Summary - Research Study

1.0 **Study Materials: As applicable to this study, attach the following:**

- **Protocol, Dissertation Proposal or Study Plan**
- **Preliminary Data**
- **Surveys, Questionnaires or other instruments to be used with study participants**
- **References**

Document Name	Document Version #
2020-05-03 end of survey thank you webpage.pdf	0.01
2020-07-17 follow up survey 1.docx	0.01
2020-12-07 focus group guide vFinal.docx	0.01
Baseline survey - section 1	0.02
Baseline survey - section 2	0.02
Baseline survey - section 3	0.02
Baseline survey - section 4	0.02
Baseline survey - section 5 (for non-healthcare workers)	0.02
Baseline survey - section 5 for healthcare workers	0.02
Baseline survey - section 6	0.02
Baseline survey questions - CHINESE translation	0.01
Baseline survey questions - KOREAN	0.01
Baseline survey questions - SPANISH translation	0.01
Baseline survey questions - TAGALOG	0.01
Baseline survey questions - VIETNAMESE translation	0.01
COVID SRAP for IRB - 2020-04-29 (v2).docx	0.01
Covid Ventilator Explainer Video SCRIPT V4 clean.docx	0.01
Survey 3 - Chinese (simplified).pdf	0.01
Survey 3 - English.pdf	0.01
Survey 3 - Korean.pdf	0.01
Survey 3 - Spanish.pdf	0.01

Survey 3 - Tagalog.pdf	0.01
Survey 3 - Vietnamese.pdf	0.01
survey landing page	0.01
Video outline	0.01
website language - CHINESE translation	0.01
website language - KOREAN	0.01
website language - SPANISH translation	0.01
website language - TAGALOG translation	0.01
website language - VIETNAMESE translation	0.01

2.0 ***Specific Aims: Indicate the purpose of the research, specifying the problems and/or hypotheses to be addressed.**

Aim 1: Assess changes in cardiovascular health behaviors and access to health care in patients with cardiovascular disease and other chronic illnesses.

Aim 2: Assess public and health care worker knowledge, priorities, and views on SRA policy.

Aim 3: Evaluate the impact of a brief educational intervention on knowledge, priorities, and views on SRA policy among the public, high-risk patients, and health care workers.

3.0 ***Background and Significance: Provide a summary of the background for this study and explain how it will contribute to existing knowledge.**

For greater than minimal risk biomedical studies, include preliminary data. If necessary, attach in Item 1.0 graphs or tables used to convey information. If there no preliminary data are available, briefly indicate why this proposed study is a reasonable starting point.

Background: COVID-19 has led to significant disruptions to usual operations of the health care system and routines of millions of people. Regular medical appointments have been preempted or converted to telemedicine, dietary changes have occurred due to food supply chain strain, and exercise regimens altered by physical distancing policies, all of which may affect chronic illness management, particularly for cardiovascular health. Additionally, anxiety about risk of developing COVID-19 and the availability of health care resources may be altering care-seeking behaviors even for serious conditions, e.g., myocardial infarctions, decompensations of heart failure, and strokes.
Critical care capacity during COVID-19 is also a matter of extreme concern, realizing hospit
al ICUs often run at 85-90% during normal respiratory virus season. Limitations in capacity, particularly mechanical ventilators, have necessitated scarce resource allocation (SRA) policies, which outline triage protocols while observing ethical principles of distributive justice, transparency, and equitability. In practice, these policies determine which patients are most likely to benefit from intensive care, and patients with chronic conditions (e.g., congestive heart failure), have a significantly heightened risk for mortality and are less likely to be prioritized in critical care allocation decisions. The uncertainty around prioritization and threat of rationing of care has generated public attention and anxiety evident in medical journals, news outlets, and on social media platforms.

The rapidity of this crisis has compelled rapid development and/or modification of SRA policies without the rigorous stakeholder engagement shown to improve acceptance of public policy by both laypersons and practitioners tasked with implementing and enforcing such policies. While some jurisdictions had pre-existing SRA policies, these policies have never been tested by a pandemic of this magnitude and many may not even know these policies exist. Two states (CA & NY), among the most affected by COVID-19, have recently developed new SRA policies, creating a unique opportunity to evaluate how these policies are understood and perceived by the patients most likely to be impacted by them (e.g., those with cardiovascular disease) and the practitioners who will implement them.

In addition to concerns about how COVID-19 patients with cardiovascular disease may be triaged, individuals with these conditions may be more susceptible to contracting the virus. Understanding their personal protective behaviors and management of cardiovascular risks under threat of COVID-19 is an epidemiologic imperative. Moreover, given the misinformation about SRAs and social behaviors affecting containment of COVID-19, there is a clear need to determine if a brief educational intervention can influence perception and knowledge about these containment and management policies.

We propose to undertake rapid recruitment of a national cohort of individuals living with cardiovascular conditions and health care practitioners to understand gaps in and evolution of knowledge and perceptions of public health and clinical efforts during this rapidly evolving pandemic; and to implement a randomized controlled trial within this cohort to test the ability of a brief intervention to improve knowledge and perceptions.

4.0 *Research Design and Methods: Describe in detail the design and methodology of the study.

Population & Recruitment:
For Aim 1, we will employ national outreach strategies via social media and partnerships with national organizations (e.g., patient advocacy organizations and professional societies) to recruit a sample of adults (ages 18 and older) from the general population. We will enrich this sample by direct recruitment efforts (via online health portal invitation) of over 300,000 patients in the Southern California Area who are cared for in the University of California at Los Angeles Health System (UCLA Health). For Aim 2, we will employ similar national outreach strategies involving professional language targeted posts on social media sites (e.g., Doximity, LinkedIn) to recruit a sample of health care providers. Additionally, this cohort will be enriched by direct recruitment efforts of health workers employed by UCLA. Cohort participants in Aims 1 & 2 from California and New York (residence determined based on ZIP code) will be randomized for Aim 3 to receive either a brief educational video or no intervention. All participants (Aims 1-3) will be invited to complete three surveys (baseline, one-month post-intervention follow-up, and four-month/post-outbreak follow-up). Participants will receive a link to a landing page that is unique to the recruitment source (social media versus e-mail versus other) in order to allow the investigators to keep track of the referral sources.

Surveys:
Participants will first be presented with an online consent form that includes language typical of a written consent and upon affirming consent will be directed to complete the baseline survey. The patient baseline survey will collect sociodemographic information (including ZIP code), chronic conditions and current health status, health behaviors, anxiety and stress levels, changes to health and care seeking behaviors (e.g., social distancing, medication adherence, care-seeking behaviors), and will include series of questions assessing perceptions and attitudes and knowledge about SRA policies and how they are implemented (e.g., values about how decisions are made, understanding of prioritization versus exclusion from critical care, age and chronic illness considerations). Questions are largely derived from the validated Behavioral Risk Factor Surveillance System questionnaires. For providers, additional questions about the characteristics of the provider will include specialty, years of practice, and trainee status. Follow-up surveys will re-administer baseline questions on knowledge, attitudes, perceptions, and personal health behaviors; follow-up surveys will additional ascertain exposure to media coverage about COVID and SRAs in the intervening windows. All surveys will be administered via Research Electronic Data Capture (REDCap) or Qualtrics system and links to complete follow-up surveys will be automatically delivered to participants via e-mail, with up to four reminders to complete, facilitating linkage between survey administration within individuals. E-mails will be stored separately from survey data in REDCap and will be permanently deleted upon study completion.

Focus groups:
Because COVID-19 is a rapidly evolving crisis and there are no pre-existing validated questions to ascertain COVID-19 perceptions and impacts, we plan to conduct a final qualitative follow-up (post-crisis) with a geographically and sociodemographically diverse selection of participants (both patients and providers) in order to obtain a more nuanced and complete examination of perceptions, attitudes, and experiences. Participants selected will be invited to participate approximately 4-6 months later (after the COVID19 crisis has slowed). Key cardiovascular health behavior themes will include experience with telemedicine, disruptions to health care

access, and general changes to health during and after COVID19 pandemic. Key SRA themes will include the method of selecting patients, concerns about equity, special considerations for disadvantaged or vulnerable populations, and perceptions of the crisis response in general. Groups will be conducted via web-conference using Zoom and recorded, transcribed, and then qualitative methods used to analyze transcripts for key themes. We plan to conduct approximately 15 focus groups of 5-10 participants each.

Social media commentary and engagement qualitative study:
The investigators will collate comments to posts about scarce resource allocation policy made in the public domain (e.g., on traditional news media articles, or on Twitter, LinkedIn, etc.) or as comments to postings for the study itself (retweets, comments on posting, etc). The comments will be collated without identifying information about who has made the comments and analyzed using standard qualitative methods to identify emerging themes.

The study team would like to note for the IRB that at this point, all instruments and interview guides have now been uploaded for review.

4.1 * **Will you be providing results of any experimental tests that are performed for the study?**

- ○ Yes - Complete Items 4.1.1 and 4.1.2
- ○ No
- ● **Not Applicable**

4.1.1 **You indicated in Item 4.1 that the research involves experimental tests. Please describe the tests, provide a rationale for providing participants with the experimental test results and explain what, how and by whom participants and their health care provider will be told about the meaning, reliability, and applicability of the test results for health care decisions.**

4.1.2 **Will tests be performed by a Clinical Laboratory Improvement Amendments (CLIA) approved lab?**
○ Yes ○ No

5.0 *Indicate how much time will be required of the subjects, per visit or contact, and in total for the study.

Surveys: Each survey will take about 20 minutes to complete. The surveys include 3 time points. The second of the 3 time points includes a <5 minute video explaining SRA policy. Total participant burden for surveys is approximately 45-60 minutes.

Focus groups: Participants will complete a ~1 hour focus group, which is optional and voluntary.

Maximum participant burden is estimated at 2 hours.

6.0 *Statistics and Data Analysis: Describe the proposed statistical procedures or descriptive analyses for the study. If applicable, indicate how the sample size was determined.

Surveys: Standard tests of association will be used to compare changes in uniform survey items across time within

patients, as well as association of survey responses with demographics.

Power: To detect a ½ standard deviation change in the number of correct knowledge items between the intervention and control arms with 90% power and a two-sided $\alpha=0.05$, 172 total participants are needed to complete follow-up. Based on our prior work with 85% trial retention and recruitment of 55 participants per day, we anticipate recruiting the necessary 204 baseline trial participants within 4 days. We anticipate that we will substantially exceed our targets to achieve desired trial power.

Focus groups and social media engagement: Qualitative methodology will be used to evaluate emerging themes. No formal statistical analysis is planned for the qualitative data, as it is intended to be explanatory or hypothesis generating to the surveys.

ID: IRB#20-000683 View: NEW 11.1 - Characteristics of the Study Population

This view has been locked by amendment(s)

Warning: Save your work at least every 15 minutes by clicking "Save" or "Continue."

Characteristics of the Study Population

1.0 ***Is this an observational or ethnographic study for which the number of participants observed or interviewed cannot be determined in advance.**

○ Yes ● **No**

2.0 **If you answered "no" to item 1.0, indicate the maximum number of study participants you hope to enroll:**

~250

3.0 **How many participants do you expect you will need to recruit, consent and/or screen to meet the target number above?**

Surveys: at least 250, focus groups: ~150

4.0 ***Indicate the specific inclusion criteria for enrollment of each of the groups of research participants in this study.**
If there are any inclusion criteria based on *gender, pregnancy/childbearing potential, race, ethnicity or language spoken*, explain the nature of and scientific rationale for the inclusions.

We will limit Focus Groups to English speakers only as we do not have the conduct focus groups in other languages. Surveys have been translated into Spanish, Chinese, Korean, Tagalog, and Vietnamese.

5.0 ***Indicate the specific exclusion criteria for each of the groups of research participants in this study.**
If there are any exclusion criteria based on *gender, pregnancy/childbearing potential, race, ethnicity or language spoken*, explain the nature of and scientific rationale for the exclusions.

None

6.0 ***How (chart review, additional tests/exams for study purposes, etc.), when and by whom will eligibility be determined?**

Participants will self-identify by responding to internet engagement, given that the recruitment posts/e-mails are in English, we expect that people who respond would be those who can read English.

ID: IRB#20-000683 View: NEW 11.2 - Characteristics of Study Population

This view has been locked by amendment(s)

Warning: Save your work at least every 15 minutes by clicking "Save" or "Continue."

Characteristics of Study Population

1.0 ***Indicate the age range of the study participants.**

Check all that apply:

- [ ] 0 to 6 years
- [ ] 7 to 11 years
- [ ] 12 to 17 years
- [ ] 17 or younger **in California** who can consent for themselves - see note below
- [ ] 17 or younger **outside California** who can consent for themselves - see note below
- [x] **18 years or older**

NOTE:

- For additional information on minors **in California** who are permitted to consent for themselves please refer to the section "Legal Exceptions Permitting Certain Minors to Consent" in the OHRPP Guidance document, Child Assent and Permission by Parents or Guardians
- For additional information on minors **outside of California** who are permitted to consent for themselves please refer to the section "Exceptions Outside of California" in the OHRPP Guidance document, Child Assent and Permission by Parents or Guardians

2.0 ***Indicate if any of the following populations/specimens will be specifically recruited/obtained for the study.**

- [x] **Adults who are competent to give informed consent**
- [ ] Adults unable to give informed consent
- [ ] Adults with diminished capacity to consent
- [ ] Fetal Tissue
- [ ] Neonates
- [ ] Participants Unable to Read, Speak, or understand English
- [ ] Pregnant Women/Fetuses
- [ ] Prisoners
- [ ] UCLA Faculty/Staff
- [ ] UCLA Students
- [ ] Wards
- [ ] **Unknown/Not Applicable**

3.0 *** Is it possible that there may be non-English speakers enrolled in this study or children whose parents are non-English speaking?**

Yes No

ID: IRB#20-000683 View: NEW 14.1 - Risks & Benefits

Warning: Save your work at least every 15 minutes by clicking "Save" or "Continue."

Risks & Benefits

Benefits

1.0 ***Are there any potential direct benefits (physical, psychological, social or other) to study participants?**

Yes No

1.1 **If yes, describe.**
Participants may find it therapeutic to share their experiences about this stressful pandemic.

2.0 ***Describe the potential benefits to society including the importance of the knowledge to be gained.**

Societal understanding of the ways that COVID19 has disrupted the ways that participants live their lives and seek medical care is important for future purposes of understanding pandemics and how to mitigate them.

Specific items about SRA policy are critical for those who seek to develop them. There is very limited data on public values and perceptions about SRA policy. Additionally, identifying knowledge gaps on what SRA policies entail and how they work is critical to modifying dissemination efforts in advance of and during pandemics and disasters.

Risks

3.0 ***Indicate the potential risks/discomforts, if any, associated with each intervention or research procedure.**

Additionally discuss any measures that will be taken to minimize risks. If data are available, estimate (a) the probability that a given harm may occur, (b) its severity, and (c) its potential reversibility. The information provided should be reflected in risks section of the informed consent documents.

If this is an exempt study and there are no risks, indicate N/A. Otherwise, please see the help text.

The questionnaires may elicit psychological distress, but the risk is felt to be minimal.

Survey respondents will be provided the following verbiage at the conclusion of the survey:
We recognize that thinking about things like this can be stressful.

If you are in crisis, please contact one of the options below for help:
◦ 911
◦ National Suicide Prevention Lifeline (available 24/7) (1-800-273-8255)
◦ To chat to someone online, visit: http://www.suicidepreventionlifeline.org/

If you have experienced emotional discomfort or distress by participation in this study and wish to discuss your concerns further, you may wish to contact one of your local services:
◦ Your local university guidance/counseling services
◦ Your local university health center
◦ Your personal health care provider

Risk/Benefit Analysis

4.0 ***RISKS/BENEFIT ANALYSIS: Indicate how the *risks to the participants are reasonable in relation to anticipated benefits*, if any, to participants and the importance of the knowledge that may reasonably be expected to result from the study:**
The minimal risk of psychologic distress is surmounted by the potential societal benefits, including by improving SRA policy to ensure it aligns with societal values and preferences.

Alternatives

5.0 ***Indicate the alternatives to participating in this study.**

Check all that apply.

- [x] **All types of studies - Choose not to participate in the study**
- [ ] Clinical/Intervention Studies - Receive standard of care instead of participating in the study
- [ ] Clinical/Intervention Studies - Medication, device, or other treatment is available off study
- [ ] Item is Not Applicable (e.g., study of existing data)
- [ ] Other

5.1 **If "other" was selected, specify.**

5.2 **If this is a clinical/intervention study:**

Describe the standard of care or activities at UCLA (or study site) that are available to prospective participants who do not enroll in this study. If not applicable to your study, state not applicable (N/A).

ID: IRB#20-000683 View: NEW 15.1 - Data & Safety Monitoring Plan

Warning: Save your work at least every 15 minutes by clicking "Save" or "Continue."

Data & Safety Monitoring Plan

1.0 ***Is a Data and Safety Monitoring Plan (DSMP) required by the funding agency or other entity?**
○ Yes ◉ **No**

ID: IRB#20-000683 View: NEW 16.1 - Payment, Costs, and Injury

Warning: Save your work at least every 15 minutes by clicking "Save" or "Continue."

Payment, Costs, and Injury

1.0 ***Indicate what the participants will receive for their participation in the study.**

Check all that apply.

- [ ] No payment will be provided
- [ ] University check
- [ ] Course Credit
- [ ] Cash
- [x] **Gift Cards/Bruincard Deposit**
- [ ] Non-Monetary Gifts or Services
- [ ] Other (including vouchers for parking)

1.1 **If you selected Non-Monetary Gifts or Services or Other, describe:**

1.2 **If you selected *Cash* and/or *Gift Cards/Bruincard Deposit* please specify the estimated total amount of money you will require to pay all participants during the length of the entire study. This information is required by UCLA Business and Finance Services (BFS), the office that will provide the cash/gift cards for payment.**
$6,250

2.0 **If study participants will receive financial or other payment for their participation in the study, please provide the following information:**

- If applicable, the amount each participant will receive and the payment schedule to be followed including whether partial payment will be provided when the participant does not complete the study.
- If there are different plans for different populations or sub-studies, specify the groups and describe the plans.
- If families or children will be involved in the research, clarify how the payments, items or services will be apportioned.

Surveys: Participants who agree to provide their e-mail address (for purposes of tracking and providing compensation) will be entered into a raffle to win one of up to 25 $100 gift cards for participation. If they complete two of the three surveys, they will get one entry. If they complete all three surveys, they will get two entries.

Participants wishing to receive a raffle entry but decline to participate in the study or provide their e-mail with their survey response can mail a postcard with their name, e-mail address, and a statement that they wish to be considered for the raffle of a gift card to the investigators' offices at 1100 Glendon Ave, Suite 850, Los Angeles, CA 90024. For those entries the investigators receive in this manner, their e-mail

addresses will be added to the pool from which recipients will be randomly selected to win one of the gift cards by using a random number generator to select from an alphabetically ordered list of e-mail addresses until up to 25 gift cards have been allocated. The approximate odds of winning a gift card are no less than 1:1000.

Gift cards will be sent electronically by e-mail to the e-mail address on file with notification that they have won the raffle.

Focus groups: Participants who agree to participate will be provided a $25 gift card for their time at the completion of the focus group.

3.0 ***Will subjects** incur **any financial obligations from participation in the study?**

- ◯ Yes
- ◉ **No**

3.1 If yes, describe:

4.0 ***Indicate below that you are familiar with UCLA policy related to treatment and compensation for injury and that you will use in the consent form for this study the appropriate UC required statement describing "Treatment and Compensation for Injury." Click here to access the UCLA policy: Treatment and Compensation for Research Related Injury.**

Note: Select **Not Applicable** if study is minimal risk.

- ◯ Agree
- ◉ **Not Applicable**

ID: IRB#20-000683 View: NEW 18.1 - Identification/Recruitment Methods

Warning: Save your work at least every 15 minutes by clicking "Save" or "Continue."

Identification/Recruitment Methods

1.0 ***How will you identify and/or recruit participants for this study.**

Check all that apply:

- [x] **Advertisements/Flyers/Information Sheet/Internet Postings**
- [x] **Direct recruitment of potential study participants (e.g., physicians talking with their own or clinic patients about the study, contact between the study team and potential subjects in person, on the phone or on the internet, etc.)**
- [ ] Random or Other Probability Sampling
- [x] **Recruitment Letters/Emails**
- [x] **Referrals (e.g., referrals from non-investigator healthcare providers, snowball sampling, participants referring other participants, etc.)**
- [ ] Review of medical records to identify potential research participants
- [ ] Review of publicly available records

- [ ] Review of other records
- [ ] Participant pool for which potential research participants have given permission for future contact
- [ ] Potential Study Participants are identified from another IRB approved study or IRB approved screening protocol
- [ ] Other

ID: IRB#20-000683 View: NEW 18.2 - Recruitment Methods

This view has been locked by amendment(s)

Warning: Save your work at least every 15 minutes by clicking "Save" or "Continue."

Recruitment Methods

1.0 Please upload copies of your recruitment materials below. This includes advertisements, flyers, internet postings, recruitment scripts and letters/emails.

Document Name	Document Version #
2020-04-10 recruitment internet postings.docx	0.02
2020-05-02 recruitment email for focus group - direct to participant.docx	0.02
2020-05-02 recruitment email for surveys - dear colleague.docx	0.01
2020-05-02 recruitment email for surveys - direct to participant.docx	0.02
2020-05-02 reminder e-mails.docx	0.01
2020-05-07 reminder e-mails 4 mo.docx	0.01
2020-05-18 incomplete response email.docx	0.01
2020-06-08 reminder e-mail SPANISH	0.01
2020-06-08 reminder e-mails 4 mo SPANISH	0.01
2020-06-08 reminder emails 4 mo VIETNAMESE	0.01
2020-06-08 reminder emails 4 wk CHINESE	0.01
2020-06-08 reminder emails 4 wk VIETNAMESE	0.01
2020-06-08 reminder e-mials 4 mo CHINESE	0.01
2020-06-11 reminder e-mails 4 mo KOREAN	0.01
2020-06-11 reminder emails 4 mo TAGALOG	0.01
2020-06-11 reminder e-mails 4 wk KOREAN	0.01
2020-06-11 reminder emails 4 wk TAGALOG	0.01
2020-06-23 recruitment flyers (all languages)	0.05
2020-07-17 additional web advertising	0.02
MyChart Research Recruitment Message _UC COVID Study.docx	0.01

Ads/Flyers/Info Sheets/Internet Postings

2.0 If you have indicated that study participants will be recruited with advertisements/flyers (Section 18.1/Item 1.0), please indicate the type of media that will be used (e.g., newspaper, radio, internet, etc.) and/or where information will be posted or distributed.

Internet based recruitment will be the primary methodology, using Twitter, Facebook, and other social media tools.

The investigators will promote the study by posting the survey link to their own personal and professional networks using their personal and UCLA-related accounts. These may be forwarded by the contacts of the investigators to the contacts' networks and perpetuated through word of mouth/snowball sampling of posts to other contacts. Additionally, the investigators are looking into purchasing advertising to send the survey link as an advertisement to

users of these platforms.

The investigators will also use contacts in the local UC media offices to amplify the posts by using official UC social media accounts through the various campuses to retweet and promote the study.

Direct Recruitment

3.0 **If you have indicated that participants will be recruited through direct contact (Section 18.1/Item 1.0), please provide the following information:**

- A description of how, when, and where initial contact would be made (e.g. in a public setting, in a waiting room, via a phone call, via a letter, via the internet, etc.)
- If applicable to the study, indicate how the potential research participant's privacy will be maintained.
- Who will make the contact (e.g. the investigator, a patient's physician, etc.)

The study team will use their professional networks and internet based social media profiles (e.g., Twitter accounts) to disseminate the link to the study survey. Study team will use sample posts (see sample internet posting attachment) for word of mouth dissemination as well as paid advertising to promote those posts on the various platforms.

The investigators will also use contacts in the local UC media offices to amplify the posts by using official UC social media accounts through the various campuses to retweet and promote the study.

3.1 **If you will be directly recruiting potential participants who are your patients, students, laboratory workers or any others with whom you have a relationship of authority or unequal power, describe what measures you will put in place to avoid those approached from feeling pressured or unduly influenced to participate in the study.**
The investigators will not know who has and has not completed the study.

Recruitment Letters/Emails

4.0 **If you have indicated that recruitment letters will be distributed to participants (Section 18.1/item 1.0), please indicate who will send out the recruitment letter (i.e. will it be the investigator or other persons who have authorized access to the information), how inquiries will be handled, and if there will be follow-up contacts.**
E-mails will be sent to UCLA Health patients via the MyUCLAHealth portal if the study team is able to gain access to this tool from the appropriate parties. The investigators will not need entry into individual patient charts. Instead, the investigators would use the portal to send the e-mail recruitment script (email for surveys) attached above to all UCLA Health patients that have an active MyUCLAHealth portal account. The e-mail will contain the survey link to participate in the study if the patient wishes to participate.

The investigators note that we do not yet have approval from the MyUCLAHealth portal research team to use this method, and will only use this methodology if we are able to secure approval.

E-mails will be sent to UCLA employees if the study team is able to gain access to this tool from the appropriate parties. This would be done by requesting an e-mail be sent to the UCLA employee listservs (e.g., Department of Medicine Faculty).

Direct e-mail to UC constituents (faculty, staff, students, and/or patients) will be done if agreements can be worked out with each individual site, with the support of UCOP and UC Health executive leadership.

E-mails with recruitment scripts will be sent to patient advocacy groups and professional societies to forward to their members if they are interested. The investigators will not email the potential participants directly, but rather inquire with the media relations officers who control the e-mails their members receive if they would be willing to forward the link to the survey with their e-mail communications with their members. The investigators have relationships with these organizations through their professional networks.

Referrals

5.0 **If you have indicated that study participants will be identified from referrals (Section 18.1/item 1.0), please indicate the source of the referral (e.g., friends, other participants, healthcare providers) and how the referral will be elicited.**
The survey links embedded in posts and emailed are available to be forwarded via e-mail or internet posting by participants to friends or other contacts.

Research Participant Pools/Recruitment Databases

6.0 **If you have indicated that subjects will be identified and recruited from a subject pool(s) or recruitment database, (Section 18.1/item 1.0), please indicate the name of the Pool or Recruitment Database and UCLA Department. If the Pool or Recruitment Database is not at UCLA, identify the location.**
N/A

ID: IRB#20-000683 View: NEW 19.1 - Eligibility Screening

Warning: Save your work at least every 15 minutes by clicking "Save" or "Continue."

Eligibility Screening

1.0 ***Will you be conducting a preliminary assessment with potential research participants to determine study eligibility during the recruitment process?**
○ Yes ● **No**

ID: IRB#20-000683 View: NEW 20.1 - Informed Consent Process

Warning: Save your work at least every 15 minutes by clicking "Save" or "Continue."

Informed Consent Process

You indicated that adults (and/or minors who are permitted to consent for themselves) are participating in the study (Section 11.2/item 1.0 or Section 12.2/item 1.0).

For additional information on minors who are permitted to consent for themselves please refer to the section "Legal Exceptions Permitting Certain Minors to Consent" in the OHRPP Guidance document, Child Assent and Permission by Parents or Guardians.

1.0 ***Indicate your plans for obtaining informed consent for this study.**

Check all that apply:

- [ ] **Signed consent** will be obtained from the research participant or Legally Authorized Representative.
    - Signed consent means research participants will be asked to **sign and date** a written consent form.
- [x] **A waiver of signed consent is requested for the entire study. One of the following procedures will be conducted:**
    - **A written information sheet will be used. Signed consent will not be obtained from research participants.**
    - **Oral consent will be obtained from the research participant or Legally Authorized Representative (LAR)**
    - **This option should be selected if the study involves consenting participants via the internet.**
- [ ] A **waiver of consent** is being requested.
    - Research participants will **not** be asked to sign a consent form or give oral consent
- [ ] Consent will be obtained by a collaborating institution.

1.1 **- If you checked more than one plan above, list the study groups and the plan that you will use for each.**
- If you checked "Consent will be obtained by a collaborating institution", explain the consent process and upload a copy of the most recent approved consent document in item 1.2.

1.2 **If applicable, attach the consent document(s) from collaborating institution(s).**

Document Name	Document Version #
There are no items to display	

ID: IRB#20-000683 View: NEW 20.2 - Waiver of Signed Informed Consent (Consent Without a Signature)

Warning: Save your work at least every 15 minutes by clicking "Save" or "Continue."

Waiver of Signed Informed Consent (Consent Without a Signature)

You indicated that you are obtaining oral consent for the study (Section 20.1/item 1). Please provide the following information.

1.0 *__Indicate the reason that you are requesting to conduct an oral consent process instead of obtaining signed consent.__**

- (●) **The research is minimal risk and does not involve any procedures for which written consent is normally required outside the research setting (e.g., in everyday life written consent is not needed for minimal risk surveys, non-invasive health measurements, etc.) (45 CFR 46.117)**
- ( ) The only record linking the participants and the research would be the consent document, and the main risk of research would be a breach of confidentiality (45 CFR 46.117).

e.g., Participants could suffer from social stigma, embarrassment, or other harms if it became known

that they participated in research that identified them as having issues including, but not limited to, risky sexual behaviors, HIV, or mental health problems.

- [ ] If the subjects or legally authorized representatives are members of a distinct cultural group or community in which signing forms in not the norm, that the research presents no more than minimal risk of harm to subjects and provided there is an appropriate alternative mechanism for documenting that informed consent was obtained.

If you indicated that the **main risk is a breach of confidentiality**, answer 1.1, if apppropriate.

1.1 **According to DHHS regulations at 45 CFR 46.117 when the main risk of the research would be a breach of confidentiality and an oral consent process is used, each subject should be asked whether he/she wants documentation linking the subject with the research and the subject's wishes will govern.**

Check here if you want the IRB to consider allowing a waiver of this regulation so that you do not need to ask each subject if he/she wishes documentation.

- [ ] Request to waive documentation linking the participant with the research

2.0 **If the oral consent process applies only to certain parts of the study (e.g., specific procedures or populations), explain.**

ID: IRB#20-000683 View: NEW 20.3 - Description of the Consent Process

This view has been locked by amendment(s)

Warning: Save your work at least every 15 minutes by clicking “Save” or “Continue.”

Description of the Consent Process

1.0 ***Indicate the type of setting(s) in which the consent process will be conducted.**

Check all that apply.

- [ ] In a private home
- [ ] In a private room
- [ ] In a waiting room
- [ ] In a public setting
- [ ] In a group setting
- [x] **On the internet**
- [ ] Over the telephone
- [ ] Other

1.1 If you checked more than one response, or indicated other, describe.

1.2 If the setting is not private, describe the measures to protect confidentiality or indicate "not applicable."

2.0 *Indicate the measures that will be taken to provide prospective research participants with sufficient opportunity to consider whether or not to participate in the study.

Check all that apply.

- [ ] Member(s) of the study staff will meet with the prospective participants/families to review the consent document(s) and/or provide an oral explanation of the study. Individuals will be given a chance to ask questions before making a considered decision about whether or not to participate in the study.
- [ ] Prospective participants/families will have the opportunity to take the consent form(s) home and may discuss the documents with others prior to deciding whether or not to participate in the study.
- [x] **Prospective participants will self-administer the consent and send it back if they decide to participate in the study.**
- [ ] Other

2.1 If you indicated other, describe.

3.0 *Indicate the length of time subjects are given to decide whether they wish to participate in the study.
The time to participate is open-ended. Participants can take as much time is necessary to review the consent script at the beginning of the survey before deciding to proceed.

4.0 *How will you assess whether subjects understand the information conveyed during the consent process?

Check all that apply.

- [ ] Use the Subject Comprehension Tool form for research
- [ ] Investigator or study team member will evaluate during the consent process
- [ ] Other
- [x] **Not Applicable**

4.1 If you indicated other, describe.

5.0 *Attach copies of the informed consent documents, information sheets, consent scripts as applicable to this study. Include copies of translated forms, if applicable.

Document Name	Document Version #
2020-05-02 IC script for surveys.docx	0.05
2020-05-03 IC script for focus groups.docx	0.05
2020-06-08 IC script for surveys CHINESE	0.01
2020-06-08 IC script for surveys SPANISH	0.01
2020-06-08 IC Script for surveys VIETNAMESE	0.01
2020-06-11 IC Script for surveys KOREAN	0.01

2020-06-11 IC Script for surveys TAGALOG 0.01

ID: IRB#20-000683 View: NEW 22.1 - Cultural Considerations

Warning: Save your work at least every 15 minutes by clicking "Save" or "Continue."

Cultural Considerations

The following items are designed to acquaint the IRB with cultural features of the population that you are studying that may require procedures to ensure truly informed consent.

1.0 ***Check all that apply to the population(s) with which this study will be conducted.**

- [ ] Participants may be illiterate or insufficiently literate to be able to comprehend a conventional written informed consent form.
- [ ] The participants may be reluctant or unwilling to sign a written informed consent form.
- [ ] The husbands make decisions for their wives.
- [ ] Elders make decisions for younger adult family members.
- [ ] Elders make decisions for their community.
- [ ] It is considered impolite to refuse a request.
- [ ] People are fearful of refusing requests that they regard as coming from authorities.
- [x] **None of the above are applicable to this study.**

1.1 **If any of the above items are applicable to this study, indicate the steps that you will take to ensure voluntary participation after providing the study information, and if applicable, any planned involvement with the community regarding the consent process.**

ID: IRB#20-000683 View: NEW 22.2 - Non-English Speaking Study Participants

This view has been locked by amendment(s)

Warning: Save your work at least every 15 minutes by clicking "Save" or "Continue."

Non-English Speaking Study Participants

You indicated that you would involve non-English speaking participants in the study (Section 11.2/Item 2.0) and/or that there is a possibility that non-English speaking participants may be enrolled in the study (Section 11.2/Item 3.0). Please provide the following information.

1.0 ***Indicate the method that you use to conduct the consent process[1] with participants who do not speak English.**

Check all that apply.

- [x] **The consent form and other study documents will be available in the participants' primary language. Study personnel (or qualified translators) able to discuss the participation in the patients' language**

will be present for the consent process.

- [ ] Study staff or qualified translators will discuss the study in the participants' language.
- [ ] An oral consent process will be used. Study personnel (or qualified translators) able to discuss the participation in the participants' language will be present for the consent process.
- [x] **The short form or another method will be used to conduct the consent process.**

Important Note: The short form may be used in very limited circumstances. For additional information please refer to the " 'Short Form' Method" section of the OHRPP guidance document, Research Involving Non-English Speaking Research Participants.

1.1 **If you checked "short form or another method", provide additional details.**
As with English speakers, we are providing an online informed consent process whereby participants are provided with the consent document online in their preferred language.

2.0 *__How will you maintain the ability to communicate with non-English speakers throughout their participation in the study?__
Indicate "N/A" if not applicable to your study.
Russell Buhr (PI) is fluent in Spanish and will be available to discuss with Spanish speakers if necessary. For additional languages, we will use UCLA Health certified translation services as needed for verbal communication with subjects.

3.0 ***If you are conducting research for which there is a real or foreseeable risk of biomedical harm in the state of California, indicate your agreement that you will provide the participants who do not read, speak, or understand English a copy of the Research Participants Bill of Rights in a language in which they are fluent. Translations into the most common languages in the greater Los Angeles area are available for download on the OHRPP website.**

- ( ) Agree
- (•) **Not Applicable**

[1] *If minors are involved in the study, this would also include the processes of obtaining parental permission and assent, as applicable.*

ID: IRB#20-000683 View: NEW 24.0 - Additional Information and/or Attachments

This view has been locked by amendment(s)

Warning: Save your work at least every 15 minutes by clicking "Save" or "Continue."

Additional Information and/or Attachments

1.0 **Attach any other documents that have not been specifically requested in previous items, but are needed for IRB Review.**

Document Name	Document Version #
Mangino_HIPPA training certificate.pdf	0.01
Mangino-CITI-Biomedical 101.pdf	0.01
Mangino-CITI-CITI Good Clinical Practice.pdf	0.01
Mangino-CITI-Responsible Conduct of Research.pdf	0.01
Mangino-CITI-Social & Behavioral Educational Modules.pdf	0.01

study logo 0.01

2.0 If there is any additional information that you want to communicate about this study, include it in the area provided. Note: this section should not be used instead of the standard application items.

The study team would like to note for the IRB that at this point, all survey instruments and focus group guides have been uploaded for approval.

Study is registered with ClinicalTrials.gov: NCT04373135

ID: IRB#20-000683 View: NEW 100.0 - Instructions for Study Submission

Instructions for Study Submission

You have completed your application, **but it has *not yet been submitted*.**

FOLLOW THESE STEPS TO SUBMIT THE APPLICATION TO THE IRB FOR REVIEW:

1. Click the **Finish** button to return to exit the SmartForm and return to the study workspace.
2. Use the **View SmartForm Progress** function to make sure that the application is complete.
3. If you are the PI or PI Proxy, click **Submit Study** under **My Activities**. If you are a member of the study team, you can let the PI know that the study is ready to submit by clicking **Send Ready Notification.**
4. Once the study is submitted, the state indicator at the top of the page will no longer display **Pre-Submission.**
5. After submission of the study, the **PI Assurances** activity will immediately become available under **My Activities**. The PI should provide his/her assurances at that time. If the PI is not available, the study can be submitted by a PI Proxy and the assurances provided at a later time. The study will be reviewed by the IRB while the **PI Assurances** are pending; however, it will not be approved until the **PI assurances** are completed.
6. ***If there is a Faculty Sponsor for the study***: The study can not be submitted to the IRB until the Faculty Sponsor provides his/her assurances through **FS Assurances** activity.

ID: IRB#20-000683 View: Display - Method Description

Audio, Visual or Digital Recordings

Click "OK" below to return to the SmartForm page where you can select the appropriate response.

ID: IRB#20-000683 View: Display - Method Description

Certificate of Confidentiality for research not supported by NIH (please see Quick Guide and Tip Sheet)

The Certificate of Confidentiality button in this section is only if your study is NOT supported or conducted by NIH but you will obtain a Certificate of Confidentiality (for example, for studies collecting information about

illegal drug use).

If you previously checked this box for an NIH-supported study before the policy change, you do not need to change your response here.

Certificates of Confidentiality are issued by the National Institutes of Health (NIH) to protect the privacy of research subjects by protecting investigators and institutions from being compelled to release information that could be used to identify subjects with a research project. Certificates of Confidentiality are issued to institutions or universities where the research is conducted. They allow the investigator and others who have access to research records to refuse to disclose identifying information in any civil, criminal, administrative, legislative, or other proceeding, whether at the federal, state, or local level.

Effective October 1, 2017, NIH has updated its policy for issuing Certificates of Confidentiality for NIH-funded and conducted research. For information about the policy change or about obtaining Certificates for research supported by other agencies, please see https://humansubjects.nih.gov/coc/index.

Click "OK" below to return to the SmartForm page where you can select the appropriate response.

ID: IRB#20-000683 View: Display - Method Description

Clinical Trial of a Drug, Biologic, Device or a Behavioral Intervention (complete item 3.0 below)

A clinical trial is a research study designed to answer specific questions about medical or behavioral treatments. The trial may be interventional or observational. Interventional studies are those in which the research participants are assigned by the investigator to a treatment or other intervention, and the outcomes measured. Observational studies are those in which individuals are observed and the outcomes are measured by the investigators.

Click "OK" below to return to the SmartForm page where you can select the appropriate response.

ID: IRB#20-000683 View: Display - Method Description

Community Based Research

Click "OK" below to return to the SmartForm page where you can select the appropriate response.

ID: IRB#20-000683 View: Display - Method Description

Controlled Substances (Schedule I or II)

Check here only if you are using a Schedule I or II Controlled substance in this study. Research using Schedule I or Schedule II controlled Substances must be submitted to the Research Advisory Panel of California for review and approval prior to initiation. Research using Schedule III, IV, or V Controlled Substances as a study drug do not require review by the Research Advisory Panel. For further information see: http://ag.ca.gov/research/guide.php o Schedule I Controlled Substances are drugs or substances with a high potential for abuse, that have no currently accepted medical use in treatment in the United States. Examples of Schedule I Controlled Substances are: heroin, lysergic acid diethylamide (LSD), methylenedioxy-methamphetamine (MDMA), marijuana, and psilocybin. o Schedule II Controlled Substances are drugs or substances with a high potential for abuse, that have a currently accepted medical use in treatment in the United States, or a currently accepted medical use with severe restrictions. Examples of Schedule II Controlled

Substances are: fentanyl, methadone, methylphenidate, morphine, and oxycodone. For further information see: http://www.deadiversion.usdoj.gov/schedules/index.html

Click "OK" below to return to the SmartForm page where you can select the appropriate response.

ID: IRB#20-000683 View: Display - Method Description

Deception or Partial Disclosure

Deception includes withholding information about the real purpose of the study or purposely giving subjects false information about some aspect of the research to prevent bias. Some professions, such as the American Psychological Association (APA) have ethical codes regarding the use of deception in research. (See sections 8.07 and 8.08 at http://www.apa.org/ethics/code/index.aspx#807) If deception is included in the study, you must also apply for approval of a waiver of the informed consent process (Section 20.1) in addition to selecting the other consent procedures planned for the study (e.g., written or oral consent).

Click "OK" below to return to the SmartForm page where you can select the appropriate response.

ID: IRB#20-000683 View: Display - Method Description

Devices/Diagnostics (Note: Submit all HUDs in BruinIRB)

A medical device is defined, in part, as any health care product that does not achieve its primary intended purposes by chemical action or by being metabolized. Medical devices include, among other things, surgical lasers, wheelchairs, sutures, pacemakers, vascular grafts, intraocular lenses, and orthopedic pins. Medical devices also include diagnostic aids such as reagents and test kits for in vitro diagnosis (IVD) of disease and other medical conditions such as pregnancy. For further information see: http://www.fda.gov/oc/ohrt/irbs/irbreview.pdf

Click "OK" below to return to the SmartForm page where you can select the appropriate response.

ID: IRB#20-000683 View: Display - Method Description

Drugs/Biologics/Dietary Supplements

- Drug: The term "drug" means: articles recognized in the official United States Pharmacopoeia, official Homoeopathic Pharmacopoeia of the United States, or official National Formulary, or any supplement to any of them; and articles intended for use in the diagnosis, cure, mitigation, treatment, or prevention of disease in man or other animals; and articles (other than food) intended to affect the structure or any function of the body of man or other animals.
- Biologics vs. Drugs: Most drugs consist of pure chemical substances and their structures are known. Most biologics, however, are complex mixtures that are not easily identified or characterized. Biological products differ from conventional drugs in that they tend to be heat-sensitive and susceptible to microbial contamination. This requires sterile processes to be applied from initial manufacturing steps. For more information see: http://www.fda.gov/consumer/updates/biologics062608.html#drugs
- Dietary Supplements are products that are intended to supplement the diet and have one of the following ingredients:
    - ☐ A vitamin
    - ☐ A mineral
    - ☐ An herb or other botanical
    - ☐ An amino acid

- ☐ A dietary substance for use by man to supplement the diet by increasing the total daily intake
- ☐ A concentrate, metabolite, constituents, or an extract of combinations of these ingredients.

For additional information see: http://www.foodsafety.gov/~dms/supplmnt.html

Click "OK" below to return to the SmartForm page where you can select the appropriate response.

ID: IRB#20-000683 View: Display - Method Description

Genetic Analyses/Genotyping

Genetic analyses/genotyping include, but are not limited to, studies of inheritable conditions or traits, gene markers or mutations, and pedigrees.

Click "OK" below to return to the SmartForm page where you can select the appropriate response.

ID: IRB#20-000683 View: Display - Method Description

Human Embryonic Stem Cells and/or Induced Pluripotent Stem Cells

Research with human embryonic stem cells (hESC) and related lines requires IRB review under the following conditions: o Clinical research in which human subjects are given hESCs or related products. o When the UCLA research team will have a research related direct interaction or intervention with the cell donors, including donation of blastocysts or gametes for the purpose of creating hESCs,. o Cells provided to the UCLA research team that have identifiers or codes that can be linked back to the donor. Research involving hESC requires review and approval by the ESCRO Committee. For further information see: http://www.stemcell.ucla.edu/research

Click "OK" below to return to the SmartForm page where you can select the appropriate response.

ID: IRB#20-000683 View: Display - Method Description

Human Gene Transfer/ Recombinant DNA

Studies involving gene transfer and/or recombinant DNA require approval of the UCLA Institutional Biosafety Committee (IBC). Human gene transfer is an investigational method for correcting defective genes responsible for disease development through one of the following techniques: o A normal gene may be inserted into a nonspecific location within the genome to replace a nonfunctional gene. o An abnormal gene could be swapped for a normal gene. o The abnormal gene could be repaired through selective reverse mutation, which returns the gene to its normal function. o The regulation of a particular gene could be altered. Recombinant DNA molecules, according to the NIH Guidelines, are defined as either: (i) molecules that are constructed outside living cells by joining natural or synthetic DNA segments to DNA molecules that can replicate in a living cell, or (ii) molecules that result from the replication of those described in (i) above.

Click "OK" below to return to the SmartForm page where you can select the appropriate response.

ID: IRB#20-000683 View: Display - Method Description

Infectious Agents

Studies involving the use of Risk Group 2 or 3 infectious agents (such as bacteria, fungi, parasites, prions, rickettsia, viruses, etc.) require approval of the UCLA Institutional Biosafety Committee (IBC).

Click "OK" below to return to the SmartForm page where you can select the appropriate response.

ID: IRB#20-000683 View: Display - Method Description

Non-FDA approved medical equipment used with UCLA hospital patients or research participants that operate under the UCLA Hospital License.

Clinical Engineering is responsible for completing incoming inspections on investigational devices that are used to diagnose, treat or monitor a patient and that are used in the patient care area on site at UCLA, but *not* in other hospitals such as Cedars Sinai, CHLA, or Drew. If a device is FDA and/or testing - laboratory approved for the purpose it was designed, then evaluation is not required of the device. If you have a copy of an inspection report from Clinical Engineering, please attach here. As appropriate, please contact Clinical Engineering at 310-267-9000 to arrange an inspection.

Click "OK" below to return to the SmartForm page where you can select the appropriate response.

ID: IRB#20-000683 View: Display - Method Description

Radiation (Standard of Care or Investigational Use of radioactive materials, radiation producing machines or ionizing radiation)

Note: This includes CT-guided biopsy, fluoroscopy use, etc.; MRI is not included. The radiological procedures included in this study must be described in the SafetyNet system. Please create a new SafetyNet application after submitting this webIRB application to the IRB for review.

Click "OK" below to return to the SmartForm page where you can select the appropriate response.

ID: IRB#20-000683 View: Display - Method Description

Substance Abuse Research (with Medication)

Research for the treatment of controlled substance addiction or abuse that uses any drug (scheduled or not) as treatment, requires the review and approval of the Research Advisory Panel of California prior to initiation. For further information see: For further information see: http://ag.ca.gov/research/guide.php

Click "OK" below to return to the SmartForm page where you can select the appropriate response.

ID: IRB#20-000683 View: Display - Method Description

Treatment in an Emergency Setting (with request to waive consent)

Federal regulations allow certain research activities to be conducted in emergency settings with waiver of informed consent - in the interest of facilitating potentially life-saving and life-enhancing research with protecting the rights and welfare of participants. For further information see: o OHRP Guidance:

http://www.hhs.gov/ohrp/humansubjects/guidance/hsdc97-01.htm o FDA Guidance: http://www.fda.gov/oc/ohrt/irbs/except.html

Click "OK" below to return to the SmartForm page where you can select the appropriate response.

ID: IRB#20-000683 View: Display - Method Description

None of the above

Click "OK" below to return to the SmartForm page where you can select the appropriate response.
