# Supplementary material for: Promotion of Knowledge and Trust Surrounding Scarce Resource Allocation Policies: A Randomized Clinical Trial
Source: JAMA Health Forum. 2024 Oct 18;5(10):e243509. doi: 10.1001/jamahealthforum.2024.3509 (PMC11489882; doi:10.1001/jamahealthforum.2024.3509)
Supplement: Supplement 2. — eFigure 1. Distribution of timing of survey responses eFigure 2. Days between survey administrations eFigure 3. Marginal estimates of percentage of correct responses to knowledge items by randomization group and time point by domain eTable 1. Questions from SRA survey with sampling universe eTable 2. Individual item mean scores by treatment group and time eTable 3. Marginal estimates for correct knowledge responses (main analysis) eTable 4. Marginal estimates for % correct knowledge responses (main analysis) eTable 5. Marginal estimates for trust and anxiety items (main analysis) eTable 6. Marginal estimates for correct knowledge responses by self-reported health care professional status (sensitivity analysis) eTable 7. Marginal estimates for trust and anxiety items by self-reported health care professional status (sensitivity analysis) eTable 8. Overall knowledge improvement treatment effect – stratified analyses by demographics eTable 9. Trust and anxiety item treatment effect – stratified analyses by demographics eTable 10. Comparison of imputed and unimputed datasets on outcomes [file jamahealthforum-e243509-s002.pdf]

Supplemental Online Content

Buhr RG, Romero R, Wisk LE. Promotion of knowledge and trust surrounding scare resource allocation policies: a randomized clinical trial. *JAMA Health Forum*. 2024;5(10):e243509. doi:10.1001/jamahealthforum.2024.3509

eFigure 1. Distribution of timing of survey responses
eFigure 2. Days between survey administrations
eFigure 3. Marginal estimates of percentage of correct responses to knowledge items by randomization group and time point by domain
eTable 1. Questions from SRA survey with sampling universe
eTable 2. Individual item mean scores by treatment group and time
eTable 3. Marginal estimates for correct knowledge responses (main analysis)
eTable 4. Marginal estimates for % correct knowledge responses (main analysis)
eTable 5. Marginal estimates for trust and anxiety items (main analysis)
eTable 6. Marginal estimates for correct knowledge responses by self-reported health care professional status (sensitivity analysis)
eTable 7. Marginal estimates for trust and anxiety items by self-reported health care professional status (sensitivity analysis)
eTable 8. Overall knowledge improvement treatment effect – stratified analyses by demographics
eTable 9. Trust and anxiety item treatment effect – stratified analyses by demographics
eTable 10. Comparison of imputed and unimputed datasets on outcomes

This supplemental material has been provided by the authors to give readers additional information about their work.

© 2024 Buhr RG et al. *JAMA Health Forum.*

eFigures:

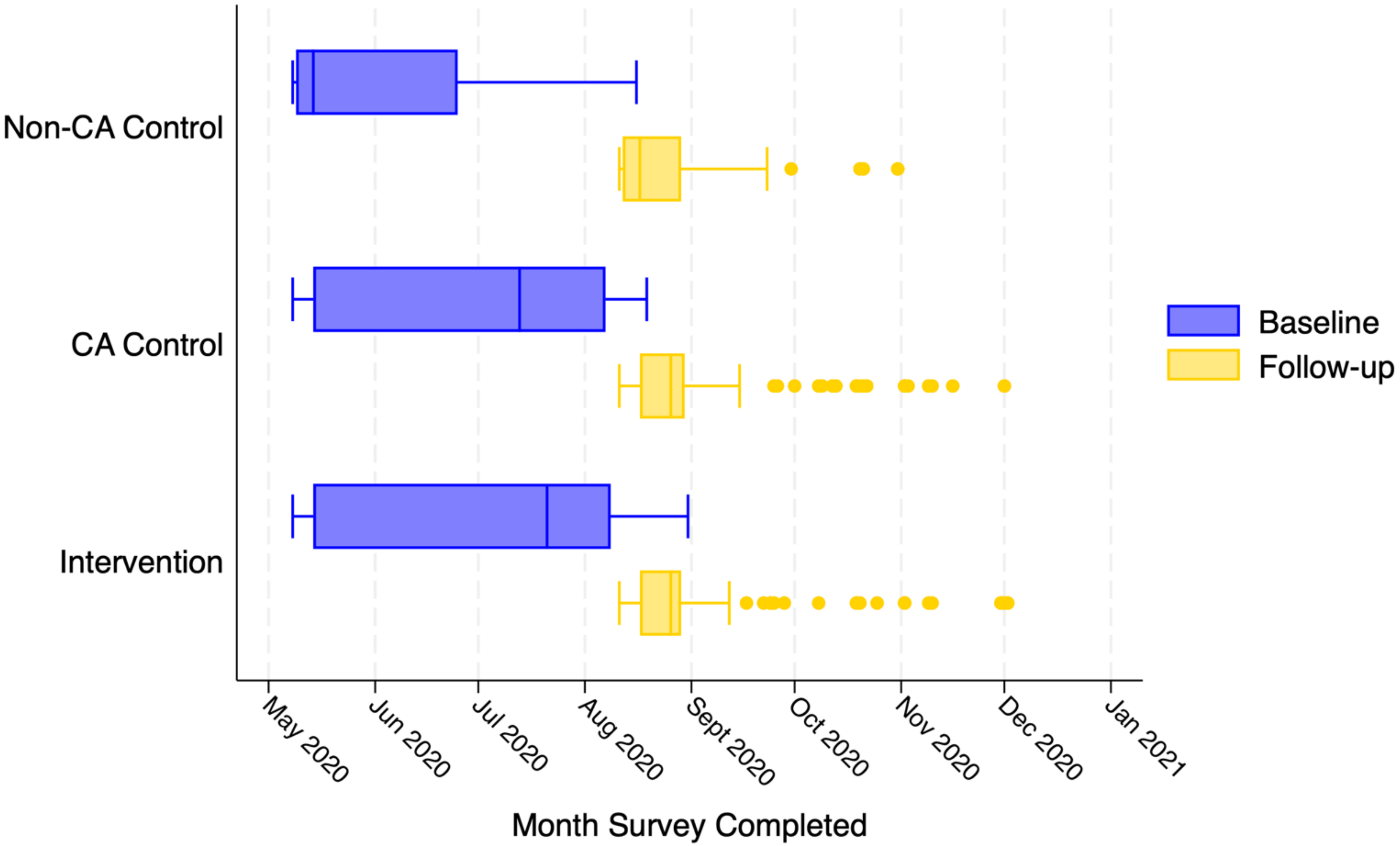

eFigure 1: Distribution of timing of survey responses

© 2024 Buhr RG et al. *JAMA Health Forum.*

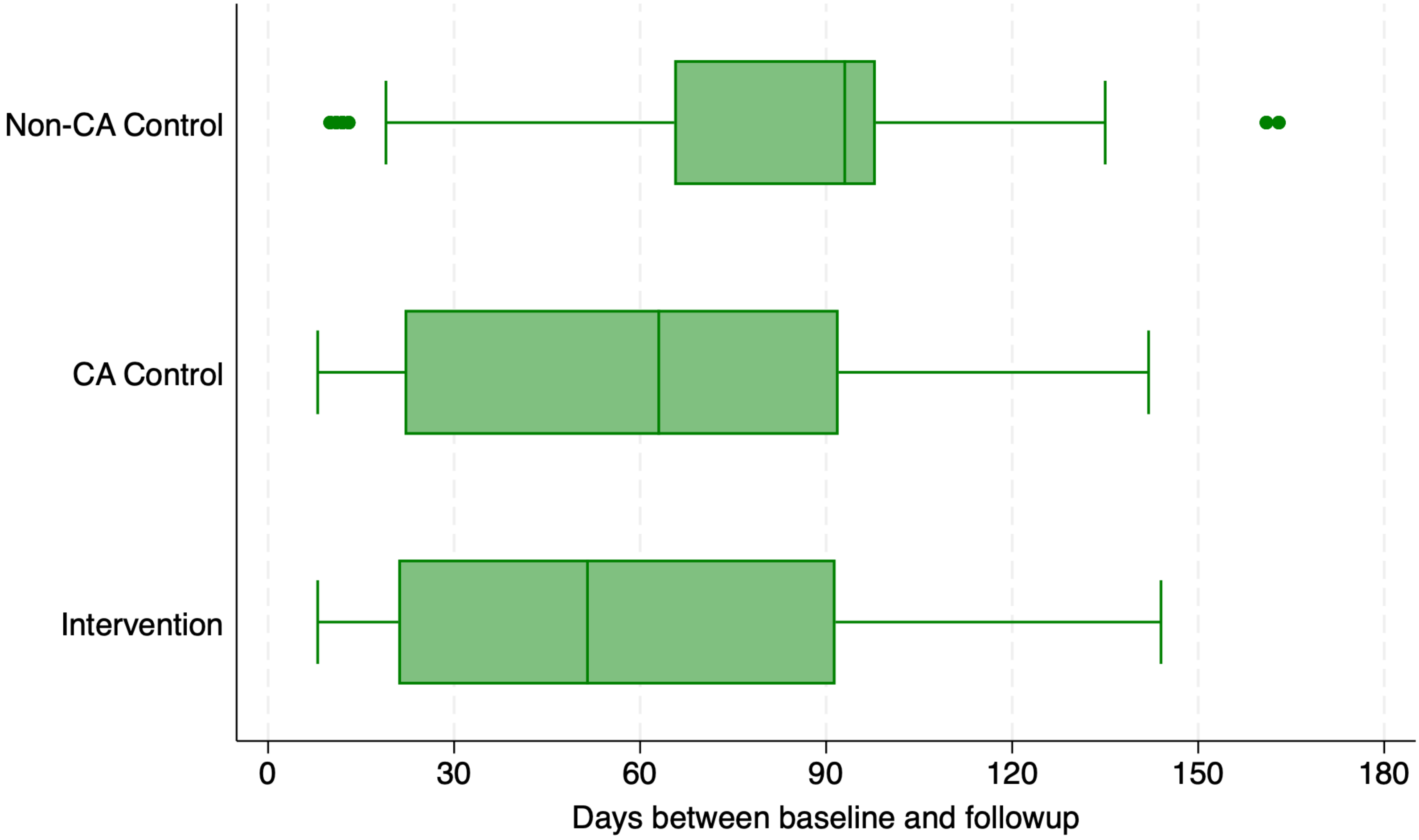

eFigure 2: Days between survey administrations

© 2024 Buhr RG et al. *JAMA Health Forum.*

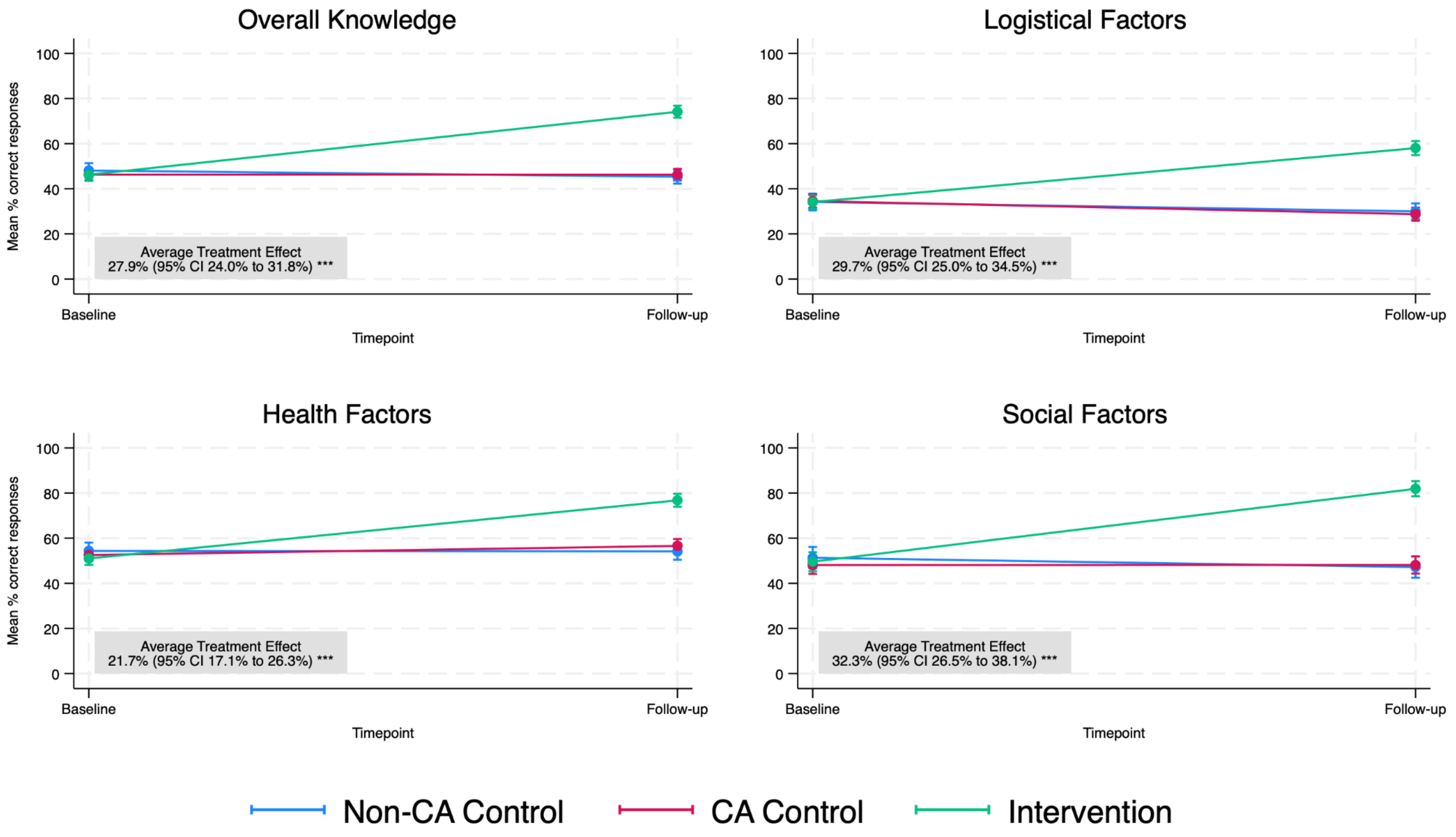

*eFigure 3: Marginal estimates of percentage of correct responses to knowledge items by randomization group and timepoint by domain. Point estimate of group mean with 95% confidence interval. Text boxes show difference in differences between intervention and control (average treatment effect) with confidence interval. * = P<0.05, ** = P<0.01, *** = P<0.001*

© 2024 Buhr RG et al. *JAMA Health Forum.*

Supplemental eTables:

eTable 1: Questions from SRA survey with sampling universe

**Section Introductory Header**			
**Universe**	**Header Text**		
**HCP Header**	**COVID and Crisis Situations.**<br>Additionally, we are interested in learning more about what you know and how you feel about the ways hospitals are preparing for surges in the number of patients coming to hospitals.<br><br>One aspect in particular we would like to ask you about is how hospitals and doctors would decide who would get critical care resources (mainly mechanical ventilators, but also other things that may be in scarce supply like dialysis or ECMO machines, supplies and medications, and staff needed for ICU care) if there weren't enough for everyone who needed one during a crisis.<br><br>The situations described would ONLY apply in situations that were so bad that the hospital or health care workers could not possibly uphold the usual standards of care, and did not have enough life-saving supplies, equipment, or staff to care for everyone who needed help.		
**Non-HCP Header**	**COVID and Crisis Situations.**<br>Additionally, we are interested in learning more about what you know and how you feel about the ways hospitals are preparing for surges in the number of patients coming to hospitals.<br>One aspect in particular we would like to ask you about is how hospitals and doctors would decide who would get life support machines (for example, mechanical ventilators or respirators, critical medications and supplies, or staff needed to operate an ICU) if there weren't enough for everyone who needed one during a crisis.<br><br>The situations described would ONLY apply in situations that were so bad that the hospital or health care workers could not possibly uphold the usual standards of care, and did not have enough life-saving supplies, equipment, or staff to care for everyone who needed help.”		
**Section 1: Knowledge surrounding logistics of SRA policy**			
**Section Scaling**	1: This if false, 2: I’m not sure, 3: This is true		
**Universe**	**Question Prompt Text**	**Proportion not answered baseline[1]**	**Proportion not answered follow-up[1]**
**HCP Header**	*How accurate do you think the following statements are about how hospitals and health care workers may make decisions in general about who would get critical care resources (such as life support/mechanical ventilators) during a crisis like a disaster or pandemic?*<br><br>*This would be a crisis so catastrophic that providing usual or standard levels of care to all patients is no longer possible, and there aren't enough critical care resources (such as ventilators, dialysis machines, ECMO circuits, other supplies, medications, or staff) to care for everyone who needs these resources, and some people will die without these resources.*	---	---
**Non-HCP Header**	*How accurate do you think the following statements are about how hospitals and health care workers may make decisions in general about who would get life support (such as a ventilator/respirator, also called a breathing machine) during a crisis like a disaster or pandemic?*<br><br>*This would be a crisis so catastrophic that providing usual or standard levels of care to all patients is no longer possible, and there aren't enough critical care resources (such as life support machines, supplies, medications, or staff) to care for everyone who needs these resources, and some people might die without these resources.*	---	---

© 2024 Buhr RG et al. *JAMA Health Forum.*

ALL	Individual doctors will make decisions on a case-by-case basis	0%	0%
ALL	Hospital committees will make decisions on a case-by-case basis	0%	0%
ALL	Hospital committees will make decisions by applying the same set of rules to everyone	0%	0%
ALL	Some patients may be taken off of life support in order to provide it to other patients who are more likely to survive	0%	0%
ALL	The same rules should apply to all patients even if they were admitted to the hospital before the crisis started	0%	0%
**Section 2: Knowledge surrounding use of health factors on resource allocation**			
**Section Scaling**	1: Would be <u>less</u> likely to get life support, 2: Would not influence decisions about life support, 3: Would be <u>more</u> likely to get life support, 4: I don't know/I'm not sure		
**Universe**	**Question Prompt Text**	**Proportion not answered baseline**	**Proportion not answered follow-up**
**HCP Header**	*How accurate do you think the following statements are about how healthcare workers and hospitals will use health factors to decide who gets critical care resources (like mechanical ventilators) during a crisis like a disaster or pandemic?*<br><br>*As a reminder, this would be a crisis so catastrophic that providing usual or standard levels of care to all patients is no longer possible, and there aren't enough critical care resources (such as ventilators, dialysis machines, ECMO circuits, other supplies, medications, or staff) to care for everyone who needs these resources, and some people will die without these resources.*	---	---
**Non-HCP Header**	*How accurate do you think the following statements are about how hospitals and health care workers may make decisions in general about who would get life support (such as a ventilator/respirator, also called a breathing machine) during a crisis like a disaster or pandemic?*<br><br>*This would be a crisis so catastrophic that providing usual or standard levels of care to all patients is no longer possible, and there aren't enough critical care resources (such as life support machines, supplies, medications, or staff) to care for everyone who needs these resources, and some people might die without these resources.*	---	---
ALL	Patients with coronavirus (COVID-19)	0.26%	0.13%
ALL	Patients who are pregnant	0.26%	0.13%
ALL	Patients with severe chronic illnesses (for example: advanced heart failure or cancer) that limit their life span	0.26%	0.13%
ALL	Patients who are deemed less likely to survive and make it out of the hospital alive	0.26%	0.13%
ALL	Patients who are elderly	0.26%	0.13%
ALL	Patients with physical disabilities (for example: being blind or using a wheelchair)	0.26%	0.13%
ALL	Patients with cognitive or intellectual disabilities (for example: autism or developmental delay)	0.26%	0.13%
**Section 3: Knowledge surrounding use of health factors on resource allocation**			
**Section Scaling**	1: Would be <u>less</u> likely to get life support, 2: Would not influence decisions about life support, 3: Would be <u>more</u> likely to get life support, 4: I don't know/I'm not sure		
**Universe**	**Question Prompt Text**	**Proportion not answered baseline**	**Proportion not answered follow-up**

© 2024 Buhr RG et al. *JAMA Health Forum.*

**HCP Header**	*Please tell us what you know about how hospitals or healthcare workers will use non-health-related factors to decide if certain groups of people get life support during a crisis like a disaster or pandemic?*<br><br>*As a reminder, this would be a crisis so catastrophic that providing usual or standard levels of care to all patients is no longer possible, and there aren't enough critical care resources (such as ventilators, dialysis machines, ECMO circuits, other supplies, medications, or staff) to care for everyone who needs these resources, and some people will die without these resources.*	---	---
**Non-HCP Header**	*Please tell us what you know about how hospitals or healthcare workers will use non-health-related factors to decide if certain groups of people get life support during a crisis like a disaster or pandemic?*<br><br>*Once again, this would be a crisis so catastrophic that providing usual or standard levels of care to all patients is no longer possible, and there aren't enough critical care resources (such as life support machines, supplies, medications, or staff) to care for everyone who needs these resources, and some people might die without these resources.*	---	---
ALL	People who are health care workers	0.78%	0.26%
ALL	People who are wealthy, famous, or in positions of power (for example: celebrities or politicians)	0.78%	0.26%
ALL	People who are a racial or ethnic minority	0.78%	0.26%
ALL	People who are LGBTQ+ (e.g., lesbian, gay, bisexual, or transgender)	0.78%	0.26%
ALL	People who are prisoners	0.78%	0.26%
ALL	People without health insurance	0.78%	0.26%
ALL	People who are undocumented immigrants	0.78%	0.26%
ALL	People who do not speak English	0.78%	0.26%
**Section 4: Trust surrounding social or non-health factors considered in SRA policy**			
**Section Scaling**	10-point Likert: **I strongly disagree with this (1)** to **(10) I strongly agree with this.** or I'd prefer not to answer		
**Universe**	**Question Prompt Text**	**Proportion not answered baseline**	**Proportion not answered follow-up**
ALL Header	*Please tell us a little more about how strongly you agree or disagree with the following statements about how policies like this would be applied, and how you feel about them in general.*	---	---
ALL	I trust that hospitals and doctors will apply policies like this in a fair and consistent way	4.42%	2.73%
ALL	I trust hospitals and doctors to be honest and transparent about how resources are used in a crisis	4.68%	2.60%
ALL	I feel anxious or worried when I think about policies like these	4.16%	2.21%
HCP Only	I feel like I would be distressed or uncomfortable if I had to carry out policies like these	9.65%	2.19%
HCP Only	I would have a moral objection to carrying out a policy like this	13.16%	4.39%
**Section 5: Feedback on Intervention (Intervention Group Only)**			
**Section Scaling**	10-point Likert: **I strongly disagree with this (1)** to **(10) I strongly agree with this,** or I'd prefer not to answer		
**Universe**	**Question Prompt Text**	**Proportion not answered baseline**	**Proportion not answered follow-up**

© 2024 Buhr RG et al. *JAMA Health Forum.*

ALL Header	*Please tell us your opinions about the video you just watched.*	---	---
ALL	This video explained things in a way that was easy to understand.	N/A	1.09%
ALL	This video clarified how resource allocation policies work.	N/A	1.09%
ALL	This video increased my anxiety about resource allocation policies.	N/A	1.09%
ALL	This video raised new or unanswered questions about these policies.	N/A	1.09%

1. Among N=770 respondents included in the analytic sample

© 2024 Buhr RG et al. *JAMA Health Forum.*

eTable 2: Individual item mean scores by treatment group and time

Item	Baseline		Follow-up		Difference-in-Differences	
	Control Arm	Treatment Arm	Control Arm	Treatment Arm	Tx Effect	
**Knowledge - Logistics**	% Correct		% Correct		Δ % Correct	P-value
Individual doctors will make decisions on a case-by-case basis	24.2%	24.3%	17.5%	63.8%	46.1%	<.001
Hospital committees will make decisions on a case-by-case basis	16.2%	14.1%	14.6%	22.8%	10.4%	0.02
Hospital committees will make decisions by applying the same set of rules to everyone	38.1%	39.5%	34.8%	75.4%	39.2%	<.001
Some patients may be taken off of life support in order to provide it to other patients who are more likely to survive	49.0%	48.2%	37.1%	71.0%	34.7%	<.001
The same rules should apply to all patients even if they were admitted to the hospital before the crisis started	45.4%	44.6%	39.7%	57.2%	18.3%	<.001
**Knowledge - Health Factors**						
Patients with coronavirus (COVID-19)	27.5%	28.8%	41.1%	72.6%	30.2%	<.001
Patients who are pregnant	79.1%	72.3%	82.1%	86.9%	11.6%	0.006
Patients with severe chronic illnesses (for example: advanced heart failure or cancer) that limit their life span	63.6%	64.2%	52.6%	63.9%	10.6%	0.03
Patients who are deemed less likely to survive and make it out of the hospital alive	79.8%	79.6%	69.9%	85.0%	15.4%	<.001
Patients who are elderly	20.5%	19.3%	28.8%	69.0%	41.4%	<.001
Patients with physical disabilities (for example: being blind or using a wheelchair)	40.1%	38.0%	60.3%	80.7%	22.5%	<.001
Patients with cognitive or intellectual disabilities (for example: autism or developmental delay)	57.3%	56.2%	61.6%	82.1%	21.6%	<.001
**Knowledge - Social Factors**						
People who are health care workers	51.5%	56.0%	59.2%	77.3%	13.6%	0.001
People who are wealthy, famous, or in positions of power (for example: celebrities or politicians)	28.4%	33.0%	29.8%	78.0%	43.7%	<.001
People who are a racial or ethnic minority	53.2%	54.6%	51.5%	84.2%	31.3%	<.001
People who are LGBTQ+ (e.g., lesbian, gay, bisexual, or transgender)	64.9%	66.7%	63.9%	89.7%	24.1%	<.001
People who are prisoners	40.1%	40.7%	39.5%	80.6%	40.6%	<.001
People without health insurance	48.5%	48.7%	44.1%	81.7%	37.3%	<.001
People who are undocumented immigrants	46.2%	46.9%	42.8%	81.7%	38.1%	<.001
People who do not speak English	55.9%	54.6%	53.5%	84.6%	32.4%	<.001

© 2024 Buhr RG et al. *JAMA Health Forum.*

eTable 3: Marginal estimates for correct knowledge responses (main analysis)

	**Marginal Estimate (95% CI*)**<br>*Number Correct Responses*	**Wald Z**<br>*P-Value**
**Overall Knowledge (out of 20)**		
Baseline, Non-CA Control	9.6 (8.7, 10.5)	<.001
Baseline, CA Control	9.3 (8.6, 9.9)	<.001
Baseline, Intervention	9.2 (8.5, 10.0)	<.001
Follow-up, Non-CA Control	9.1 (8.2, 9.9)	<.001
Follow-up, CA Control	9.3 (8.6, 9.9)	<.001
Follow-up, Intervention	14.8 (13.8, 15.9)	<.001
Average Treatment Effect (DID)	5.6 (4.8, 6.4)	<.001
**Logistical Factors (out of 5)**		
Baseline, Non-CA Control	1.7 (1.4, 2.0)	<.001
Baseline, CA Control	1.7 (1.5, 1.9)	<.001
Baseline, Intervention	1.7 (1.5, 1.9)	<.001
Follow-up, Non-CA Control	1.5 (1.3, 1.7)	<.001
Follow-up, CA Control	1.4 (1.2, 1.6)	<.001
Follow-up, Intervention	2.9 (2.6, 3.2)	<.001
Average Treatment Effect (DID)	1.5 (1.2, 1.8)	<.001
**Health Factors (out of 7)**		
Baseline, Non-CA Control	3.8 (3.4, 4.2)	<.001
Baseline, CA Control	3.7 (3.4, 4.0)	<.001
Baseline, Intervention	3.6 (3.3, 3.9)	<.001
Follow-up, Non-CA Control	3.8 (3.4, 4.2)	<.001
Follow-up, CA Control	4.0 (3.7, 4.3)	<.001
Follow-up, Intervention	5.4 (5.0, 5.7)	<.001
Average Treatment Effect (DID)	1.5 (1.0, 2.0)	<.001
**Social Factors (out of 8)**		
Baseline, Non-CA Control	4.1 (3.6, 4.7)	<.001
Baseline, CA Control	3.8 (3.4, 4.3)	<.001
Baseline, Intervention	4.0 (3.5, 4.4)	<.001
Follow-up, Non-CA Control	3.8 (3.3, 4.3)	<.001
Follow-up, CA Control	3.9 (3.4, 4.3)	<.001
Follow-up, Intervention	6.6 (5.9, 7.2)	<.001
Average Treatment Effect (DID)	2.6 (2.1, 3.1)	<.001

** Bonferroni corrected for multiple comparisons*

© 2024 Buhr RG et al. *JAMA Health Forum.*

eTable 4: Marginal estimates for % correct knowledge responses (main analysis)

	**Marginal Estimate (95% CI*)** *Percent of Correct Responses*	**Wald Z** *P-Value**
**Overall Knowledge**		
Baseline, Non-CA Control	48.1% (43.8%, 52.5%)	<.001
Baseline, CA Control	46.3% (42.9%, 49.6%)	<.001
Baseline, Intervention	46.2% (42.6%, 49.9%)	<.001
Follow-up, Non-CA Control	45.3% (41.2%, 49.5%)	<.001
Follow-up, CA Control	46.3% (42.8%, 49.7%)	<.001
Follow-up, Intervention	74.2% (70.6%, 77.7%)	<.001
Average Treatment Effect (DID)	28.0% (24.0%, 31.9%)	<.001
**Logistical Factors**		
Baseline, Non-CA Control	34.2% (29.2%, 39.1%)	<.001
Baseline, CA Control	34.6% (30.6%, 38.6%)	<.001
Baseline, Intervention	34.1% (30.2%, 38.1%)	<.001
Follow-up, Non-CA Control	30.0% (25.3%, 34.7%)	<.001
Follow-up, CA Control	28.7% (24.8%, 32.7%)	<.001
Follow-up, Intervention	58.0% (53.9%, 62.2%)	<.001
Average Treatment Effect (DID)	29.7% (25.0%, 34.5%)	<.001
**Health Factors**		
Baseline, Non-CA Control	54.4% (49.5%, 59.3%)	<.001
Baseline, CA Control	52.6% (48.9%, 56.2%)	<.001
Baseline, Intervention	51.0% (47.2%, 54.9%)	<.001
Follow-up, Non-CA Control	54.2% (49.2%, 59.2%)	<.001
Follow-up, CA Control	56.6% (52.6%, 60.7%)	<.001
Follow-up, Intervention	76.8% (73.0%, 80.7%)	<.001
Average Treatment Effect (DID)	21.7% (17.1%, 26.3%)	<.001
**Social Factors**		
Baseline, Non-CA Control	51.4% (45.0%, 57.7%)	<.001
Baseline, CA Control	48.1% (42.8%, 53.3%)	<.001
Baseline, Intervention	49.6% (43.9%, 55.2%)	<.001
Follow-up, Non-CA Control	47.2% (40.9%, 53.5%)	<.001
Follow-up, CA Control	48.1% (43.0%, 53.2%)	<.001
Follow-up, Intervention	81.9% (77.4%, 86.5%)	<.001
Average Treatment Effect (DID)	32.3% (26.5%, 38.1%)	<.001

** Bonferroni corrected for multiple comparisons*

© 2024 Buhr RG et al. *JAMA Health Forum.*

eTable 5: Marginal estimates for trust and anxiety items (main analysis)

	Marginal Estimate (95% CI*)<br>*Likert Score*	Wald Z<br>*P-Value**
**"I trust that hospitals and doctors will apply policies like this in a fair and consistent way"**	**Likert Scale (1: Strongly Disagree to 10: Strongly Agree)**	
Baseline, Non-CA Control	6.1 (5.6, 6.6)	<.001
Baseline, CA Control	6.2 (5.8, 6.6)	<.001
Baseline, Intervention	6.1 (5.7, 6.5)	<.001
Follow-up, Non-CA Control	5.9 (5.4, 6.4)	<.001
Follow-up, CA Control	6.0 (5.6, 6.4)	<.001
Follow-up, Intervention	6.5 (6.1, 6.9)	<.001
Average Treatment Effect (DID)	0.5 (0.1, 0.9)	<.001
**"I trust hospitals and doctors to be honest and transparent about how resources are used in a crisis"**	**Likert Scale (1: Strongly Disagree to 10: Strongly Agree)**	
Baseline, Non-CA Control	6.0 (5.5, 6.6)	<.001
Baseline, CA Control	6.1 (5.7, 6.6)	<.001
Baseline, Intervention	6.2 (5.7, 6.6)	<.001
Follow-up, Non-CA Control	5.6 (5.1, 6.1)	<.001
Follow-up, CA Control	5.7 (5.3, 6.1)	<.001
Follow-up, Intervention	6.4 (6.0, 6.9)	<.001
Average Treatment Effect (DID)	0.7 (0.3, 1.2)	0.002
**"I feel anxious or worried when I think about policies like these"**	**Likert Scale (1: Strongly Disagree to 10: Strongly Agree)**	
Baseline, Non-CA Control	6.3 (5.8, 6.9)	<.001
Baseline, CA Control	6.4 (6.0, 6.8)	<.001
Baseline, Intervention	6.3 (5.9, 6.8)	<.001
Follow-up, Non-CA Control	6.4 (5.8, 6.9)	<.001
Follow-up, CA Control	6.4 (6.0, 6.8)	<.001
Follow-up, Intervention	6.3 (5.9, 6.8)	<.001
Average Treatment Effect (DID)	0.0 (-0.5, 0.4)	0.85
**"I feel like I would be distressed or uncomfortable if I had to carry out policies like these" ^**	**Likert Scale (1: Strongly Disagree to 10: Strongly Agree)**	
Baseline, Non-CA Control	6.9 (6.0, 7.8)	<.001
Baseline, CA Control	7.4 (6.6, 8.2)	<.001
Baseline, Intervention	7.4 (6.6, 8.2)	<.001
Follow-up, Non-CA Control	6.8 (5.9, 7.7)	<.001
Follow-up, CA Control	7.4 (6.7, 8.2)	<.001
Follow-up, Intervention	6.9 (6.1, 7.7)	<.001
Average Treatment Effect (DID)	-0.5 (-1.4, 0.4)	0.31
**"I would have a moral objection to carrying out a policy like this" ^**	**Likert Scale (1: Strongly Disagree to 10: Strongly Agree)**	
Baseline, Non-CA Control	4.9 (3.9, 5.9)	<.001
Baseline, CA Control	5.6 (4.7, 6.4)	<.001
Baseline, Intervention	4.8 (3.9, 5.7)	<.001
Follow-up, Non-CA Control	5.1 (4.1, 6.1)	<.001
Follow-up, CA Control	5.3 (4.5, 6.2)	<.001
Follow-up, Intervention	4.2 (3.3, 5.0)	<.001
Average Treatment Effect (DID)	-0.4 (-1.5, 0.7)	0.51

** Bonferroni corrected for multiple comparisons*
^ Question only asked of self-identified health care professionals (n = 208)

© 2024 Buhr RG et al. *JAMA Health Forum.*

eTable 6: Marginal estimates for correct knowledge responses by self-reported health care professional status (sensitivity analysis)

	**Marginal Estimate (95% CI*)** *Number Correct Responses*	**Wald Z** *P-Value**
**Overall Knowledge**		
Baseline, Layperson, Non-CA Control	9.4 (8.3, 10.5)	<.001
Baseline, Layperson, CA Control	8.8 (7.9, 9.6)	<.001
Baseline, Layperson, Intervention	8.7 (7.8, 9.5)	<.001
Baseline, HCP, Non-CA Control	10.1 (8.4, 11.9)	<.001
Baseline, HCP, CA Control	10.4 (8.9, 11.9)	<.001
Baseline, HCP, Intervention	10.7 (9.1, 12.2)	<.001
Follow-up, Layperson, Non-CA Control	9.1 (8.0, 10.1)	<.001
Follow-up, Layperson, CA Control	8.8 (7.9, 9.6)	<.001
Follow-up, Layperson, Intervention	14.7 (13.4, 16.1)	<.001
Follow-up, HCP, Non-CA Control	9.1 (7.5, 10.7)	<.001
Follow-up, HCP, CA Control	10.4 (8.9, 11.9)	<.001
Follow-up, HCP, Intervention	15.1 (13.0, 17.2)	<.001
Average Treatment Effect (DID): Laypersons	6.1 (5.1, 7.0)	<.001
Average Treatment Effect (DID): HCP	4.4 (2.9, 5.9)	<.001
Moderated Avg. Treatment Effect (DDD): Net Lay vs. HCP	1.7 (-0.1, 3.4)	0.06
**Logistical Factors Knowledge**		
Baseline, Layperson, Non-CA Control	1.5 (1.2, 1.8)	<.001
Baseline, Layperson, CA Control	1.7 (1.4, 1.9)	<.001
Baseline, Layperson, Intervention	1.6 (1.3, 1.9)	<.001
Baseline, HCP, Non-CA Control	2.1 (1.5, 2.7)	<.001
Baseline, HCP, CA Control	1.9 (1.4, 2.3)	<.001
Baseline, HCP, Intervention	2.0 (1.5, 2.5)	<.001
Follow-up, Layperson, Non-CA Control	1.3 (1.0, 1.6)	<.001
Follow-up, Layperson, CA Control	1.4 (1.2, 1.6)	<.001
Follow-up, Layperson, Intervention	2.9 (2.5, 3.3)	<.001
Follow-up, HCP, Non-CA Control	1.9 (1.3, 2.4)	<.001
Follow-up, HCP, CA Control	1.5 (1.1, 1.9)	<.001
Follow-up, HCP, Intervention	2.8 (2.2, 3.4)	<.001
Average Treatment Effect (DID): Laypersons	1.6 (1.2, 2.0)	<.001
Average Treatment Effect (DID): HCP	1.2 (0.6, 1.8)	<.001
Moderated Avg. Treatment Effect (DDD): Net Lay vs. HCP	0.4 (-0.3, 1.2)	0.25
**Health Factors Knowledge**		
Baseline, Layperson, Non-CA Control	3.9 (3.4, 4.4)	<.001
Baseline, Layperson, CA Control	3.5 (3.1, 3.9)	<.001
Baseline, Layperson, Intervention	3.5 (3.1, 3.8)	<.001
Baseline, HCP, Non-CA Control	3.7 (3.0, 4.4)	<.001
Baseline, HCP, CA Control	4.1 (3.5, 4.8)	<.001
Baseline, HCP, Intervention	3.9 (3.2, 4.5)	<.001
Follow-up, Layperson, Non-CA Control	3.9 (3.4, 4.4)	<.001

	Marginal Estimate (95% CI*)<br>*Number Correct Responses*	Wald Z<br>*P-Value**
Follow-up, Layperson, CA Control	3.8 (3.4, 4.2)	<.001
Follow-up, Layperson, Intervention	5.3 (4.8, 5.8)	<.001
Follow-up, HCP, Non-CA Control	3.6 (2.9, 4.3)	<.001
Follow-up, HCP, CA Control	4.3 (3.7, 5.0)	<.001
Follow-up, HCP, Intervention	5.6 (4.8, 6.3)	<.001
Average Treatment Effect (DID): Laypersons	1.5 (1.0, 2.1)	<.001
Average Treatment Effect (DID): HCP	1.5 (0.6, 2.4)	0.001
Moderated Avg. Treatment Effect (DDD): Net Lay vs. HCP	0.0 (-1.0, 1.1)	0.95
**Social Factors Knowledge**		
Baseline, Layperson, Non-CA Control	4.0 (3.3, 4.7)	<.001
Baseline, Layperson, CA Control	3.6 (3.1, 4.1)	<.001
Baseline, Layperson, Intervention	3.6 (3.1, 4.2)	<.001
Baseline, HCP, Non-CA Control	4.3 (3.2, 5.4)	<.001
Baseline, HCP, CA Control	4.4 (3.5, 5.3)	<.001
Baseline, HCP, Intervention	4.8 (3.8, 5.8)	<.001
Follow-up, Layperson, Non-CA Control	3.9 (3.2, 4.5)	<.001
Follow-up, Layperson, CA Control	3.6 (3.1, 4.1)	<.001
Follow-up, Layperson, Intervention	6.5 (5.7, 7.3)	<.001
Follow-up, HCP, Non-CA Control	3.6 (2.7, 4.6)	<.001
Follow-up, HCP, CA Control	4.6 (3.6, 5.5)	<.001
Follow-up, HCP, Intervention	6.7 (5.4, 8.0)	<.001
Average Treatment Effect (DID): Laypersons	2.9 (2.3, 3.5)	<.001
Average Treatment Effect (DID): HCP	1.7 (0.7, 2.7)	0.001
Moderated Avg. Treatment Effect (DDD): Net Lay vs. HCP	1.2 (0.0, 2.4)	0.04

** Bonferroni corrected for multiple comparisons*

© 2024 Buhr RG et al. *JAMA Health Forum.*

eTable 7: Marginal estimates for trust and anxiety items by self-reported health care professional status (sensitivity analysis)

	**Marginal Estimate (95% CI*)** *Likert Score*	**Wald Z** *P-Value**
**"I trust that hospitals and doctors will apply policies like this in a fair and consistent way"**	**Likert Scale (1: Strongly Disagree to 10: Strongly Agree)**	
Baseline, Layperson, Non-CA Control	6.3 (5.6, 6.9)	<.001
Baseline, Layperson, CA Control	6.1 (5.6, 6.6)	<.001
Baseline, Layperson, Intervention	6.0 (5.5, 6.6)	<.001
Baseline, HCP, Non-CA Control	5.9 (4.9, 6.9)	<.001
Baseline, HCP, CA Control	6.4 (5.6, 7.2)	<.001
Baseline, HCP, Intervention	6.3 (5.4, 7.1)	<.001
Follow-up, Layperson, Non-CA Control	5.9 (5.2, 6.5)	<.001
Follow-up, Layperson, CA Control	5.8 (5.3, 6.3)	<.001
Follow-up, Layperson, Intervention	6.4 (5.8, 6.9)	<.001
Follow-up, HCP, Non-CA Control	6.1 (5.1, 7.0)	<.001
Follow-up, HCP, CA Control	6.6 (5.8, 7.4)	<.001
Follow-up, HCP, Intervention	6.8 (6.0, 7.6)	<.001
Average Treatment Effect (DID): Laypersons	0.6 (0.1, 1.1)	0.03
Average Treatment Effect (DID): HCP	0.3 (-0.5, 1.2)	0.41
Moderated Avg. Treatment Effect (DDD): Net Lay vs. HCP	0.2 (-0.7, 1.2)	0.62
"I trust hospitals and doctors to be honest and transparent about how resources are used in a crisis"	**Likert Scale (1: Strongly Disagree to 10: Strongly Agree)**	
Baseline, Layperson, Non-CA Control	6.1 (5.5, 6.8)	<.001
Baseline, Layperson, CA Control	6.0 (5.5, 6.6)	<.001
Baseline, Layperson, Intervention	6.1 (5.6, 6.7)	<.001
Baseline, HCP, Non-CA Control	5.8 (4.8, 6.8)	<.001
Baseline, HCP, CA Control	6.4 (5.5, 7.2)	<.001
Baseline, HCP, Intervention	6.3 (5.4, 7.1)	<.001
Follow-up, Layperson, Non-CA Control	5.5 (4.8, 6.2)	<.001
Follow-up, Layperson, CA Control	5.4 (4.9, 5.9)	<.001
Follow-up, Layperson, Intervention	6.3 (5.7, 6.8)	<.001
Follow-up, HCP, Non-CA Control	5.8 (4.8, 6.8)	<.001
Follow-up, HCP, CA Control	6.4 (5.5, 7.2)	<.001
Follow-up, HCP, Intervention	6.8 (6.0, 7.7)	<.001
Average Treatment Effect (DID): Laypersons	0.8 (0.2, 1.3)	<.001
Average Treatment Effect (DID): HCP	0.6 (-0.3, 1.4)	0.19
Moderated Avg. Treatment Effect (DDD): Net Lay vs. HCP	0.2 (-0.8, 1.2)	0.71
"I feel anxious or worried when I think about policies like these"	**Likert Scale (1: Strongly Disagree to 10: Strongly Agree)**	
Baseline, Layperson, Non-CA Control	6.2 (5.5, 6.9)	<.001
Baseline, Layperson, CA Control	6.4 (5.9, 7.0)	<.001

© 2024 Buhr RG et al. *JAMA Health Forum.*

	Marginal Estimate (95% CI*)	Wald Z
	Likert Score	*P-Value**
Baseline, Layperson, Intervention	6.3 (5.7, 6.9)	<.001
Baseline, HCP, Non-CA Control	6.5 (5.5, 7.6)	<.001
Baseline, HCP, CA Control	6.3 (5.4, 7.2)	<.001
Baseline, HCP, Intervention	6.5 (5.6, 7.5)	<.001
Follow-up, Layperson, Non-CA Control	6.8 (6.1, 7.5)	<.001
Follow-up, Layperson, CA Control	6.5 (5.9, 7.0)	<.001
Follow-up, Layperson, Intervention	6.3 (5.8, 6.9)	<.001
Follow-up, HCP, Non-CA Control	5.6 (4.5, 6.6)	<.001
Follow-up, HCP, CA Control	6.3 (5.4, 7.1)	<.001
Follow-up, HCP, Intervention	6.2 (5.3, 7.2)	<.001
Average Treatment Effect (DID): Laypersons	0.0 (-0.5, 0.6)	0.91
Average Treatment Effect (DID): HCP	-0.2 (-1.1, 0.6)	0.58
Moderated Avg. Treatment Effect (DDD): Net Lay vs. HCP	0.3 (-0.7, 1.3)	0.60

** Bonferroni corrected for multiple comparisons*

© 2024 Buhr RG et al. *JAMA Health Forum.*

eTable 8: Overall knowledge improvement treatment effect – stratified analyses by demographics

	Improvement in number of correct knowledge items (out of 20)				
	**Treatment Effect**	**S.E.**	**P (Wald Z)**	**95% CI**	
**Race/Ethnicity**					
Asian/Pacific Islander, non-Hispanic	6.9	1.1	<.001	4.6	9.1
Black, non-Hispanic	6.1	2.2	0.006	1.7	10.5
Hispanic/Latin	5.3	1.2	<.001	2.9	7.7
Multiracial, non-Hispanic	6.8	3.7	0.07	-0.4	13.9
White, non-Hispanic	5.3	0.5	<.001	4.4	6.3
Other, non-Hispanic	9.8	5.0	0.05	0.03	19.5
**Education**					
Less than a bachelor's degree	6.2	1.1	<.001	4.0	8.4
Bachelor's degree or higher	5.5	0.4	<.001	4.7	6.4
**Age**					
Under 35 years old	4.9	1.0	<.001	2.9	6.8
35-55 years old	5.5	0.7	<.001	4.2	6.8
Older than 55 years old	5.9	0.6	<.001	4.7	7.1

© 2024 Buhr RG et al. *JAMA Health Forum.*

eTable 9: Trust and anxiety item treatment effect – stratified analyses by demographics. Questions on distress and moral objection were only asked of HCP and insufficiently powered for stratified analyses and are omitted here.

	**I trust that hospitals and doctors will apply policies like this in a fair and consistent way**		**I trust hospitals and doctors to be honest and transparent about how resources are used in a crisis**		**I feel anxious or worried when I think about policies like these**	
	Treatment Effect (95% CI)	*P-Value*	*Treatment Effect (95% CI)*	*P-Value*	*Treatment Effect (95% CI)*	*P-Value*
**Race/Ethnicity**						
Asian/Pacific Islander, non-Hispanic	1.22 (0.06, 2.38)	0.04	1.39 (0.17, 2.61)	0.03	0.77 (-0.38, 1.92)	0.19
Black, non-Hispanic	0.22 (-2.61, 3.07)	0.87	1.22 (-1.81, 4.25)	0.43	-0.04 (-3.61, 3.52)	0.98
Hispanic/Latin	0.60 (-0.80, 2.01)	0.40	0.38 (-0.97, 1.74)	0.58	-0.70 (-3.38, 0.98)	0.41
Multiracial, non-Hispanic	-0.62 (-2.80, 1.55)	0.58	-1.12 (-2.88, 0.64)	0.21	-1.01 (-3.57, 1.55)	0.44
White, non-Hispanic	0.41 (-0.09, 0.91)	0.11	0.74 (0.22, 1.25)	0.005	-0.08 (-0.61, 0.45)	0.76
Other, non-Hispanic	1.75 (-1.72, 5.22)	0.32	-3.75 (-10.39, 2.89)	0.27	-0.50 (-1.97, 0.97)	0.51
**Education**						
Less than a bachelor's degree	0.44 (-0.70, 1.58)	0.45	0.21 (-1.18, 1.61)	0.76	-0.24 (-1.46, 0.97)	0.69
Bachelor's degree or higher	0.53 (0.08, 0.99)	0.02	0.80 (0.34, 1.26)	0.001	-0.02 (-0.50, 0.47)	0.95
**Age**						
Under 35 years old	1.44 (0.50, 2.38)	0.003	1.31 (0.41, 2.22)	0.004	-0.01 (-1.01, 0.99)	0.99
35-55 years old	0.16 (-0.52, 0.83)	0.65	0.52 (-0.19, 1.22)	0.15	-0.32 (-0.98, 0.34)	0.34
Older than 55 years old	0.46 (-0.19, 1.11)	0.17	0.67 (-.03, 1.37)	0.06	0.19 (-0.56, 0.94)	0.62

© 2024 Buhr RG et al. *JAMA Health Forum.*

eTable 10: Comparison of imputed and unimputed datasets on outcomes

	Dataset	DID Estimate	S.E.	95% CI		P-Value
**Knowledge**						
**Overall Knowledge**	Imputed	5.642	0.405	4.849	6.435	<.001
	Unimputed	5.590	0.404	4.799	6.381	<.001
	Difference: Unimputed-Imputed	-0.052	-0.001	-0.049	-0.054	---
**Logistical Factors**	Imputed	1.487	0.166	1.161	1.813	<.001
	Unimputed	1.487	0.166	1.161	1.813	<.001
	Difference: Unimputed-Imputed	0.000	0.000	0.000	0.000	---
**Health Factors**	Imputed	1.521	0.241	1.049	1.993	<.001
	Unimputed	1.520	0.240	1.048	1.991	<.001
	Difference: Unimputed-Imputed	-0.002	0.000	-0.001	-0.002	---
**Social Factors**	Imputed	2.634	0.265	2.114	3.153	<.001
	Unimputed	2.584	0.264	2.067	3.101	<.001
	Difference: Unimputed-Imputed	-0.050	-0.001	-0.048	-0.053	---
**Trust & Anxiety**						
**"I trust that hospitals and doctors will apply policies like this in a fair and consistent way"**	Imputed	0.547	0.225	0.107	0.987	0.02
	Unimputed	0.517	0.221	0.085	0.950	<.001
	Difference: Unimputed-Imputed	-0.030	-0.004	-0.023	-0.037	---
**"I trust hospitals and doctors to be honest and transparent about how resources are used in a crisis"**	Imputed	0.729	0.236	0.267	1.191	0.002
	Unimputed	0.720	0.233	0.263	1.177	0.002
	Difference: Unimputed-Imputed	-0.009	-0.003	-0.003	-0.014	---
**"I feel anxious or worried when I think about policies like these"**	Imputed	-0.018	0.239	-0.486	0.451	0.94
	Unimputed	-0.045	0.237	-0.509	0.419	0.85
	Difference: Unimputed-Imputed	-0.027	-0.002	-0.023	-0.032	---
**"I feel like I would be distressed or uncomfortable if I had to carry out policies like these"***	Imputed	-0.535	0.459	-1.435	0.364	0.24
	Unimputed	-0.462	0.454	-1.352	0.427	0.31
	Difference: Unimputed-Imputed	0.073	-0.005	0.083	0.063	---
**"I would have a moral objection to carrying out a policy like this"***	Imputed	-0.430	0.560	-1.528	0.669	0.44
	Unimputed	-0.377	0.566	-1.487	0.732	0.51
	Difference: Unimputed-Imputed	0.052	0.006	0.041	0.063	---

* Asked of HCP only

© 2024 Buhr RG et al. *JAMA Health Forum.*

© 2024 Buhr RG et al. *JAMA Health Forum.*
