# Supplementary material for: Promotion of Knowledge and Trust Surrounding Scarce Resource Allocation Policies: A Randomized Clinical Trial
Source: JAMA Health Forum. 2024 Oct 18;5(10):e243509. doi: 10.1001/jamahealthforum.2024.3509 (PMC11489882; doi:10.1001/jamahealthforum.2024.3509)
Supplement: Supplement 3. — Data sharing statement [file jamahealthforum-e243509-s003.pdf]

Data Sharing Statement

Buhr. Promotion of Knowledge and Trust Surrounding Scarce Resource Allocation Policies. *JAMA Health Forum*. Published October 18, 2024. doi:10.1001/jamahealthforum.2024.3509

Data
Additional Information: ClinicalTrials.gov registration NCT04373135.
Data available: Yes
Data types: Deidentified participant data, Data dictionary
How to access data: Upon reasonable request to the investigators and execution of a data use agreement.
When available: With publication

Supporting Documents
Document types: None

Additional Information
Who can access the data: researchers whose proposed use of the data has been approved
Types of analyses: Additional analyses
Mechanisms of data availability: With investigator support after approval of a proposal and a signed data use agreement.
